# Patient and Carer-Related Facilitators and Barriers to the Adoption of Assistive Technologies for the Care of Older Adults: Systematic Review

**DOI:** 10.2196/73917

**Published:** 2025-11-27

**Authors:** Stephen Malden, Kris McGill, Bruce Guthrie, Helen Frost, Susan D Shenkin, Adanna Ezike, Bethany Kate Bareham, Stewart W Mercer, Caroline Pearce, Cara Wilson, Ian Underwood, John Vines, Sue Lewis, Amy O'Donnell

**Affiliations:** 1 Scottish Collaboration for Public Health Research and Policy, School of Health in Social Science, University of Edinburgh, Old Medical School, Teviot Place, Edinburgh, United Kingdom, 44 0131651 ext 3969; 2 Advanced Care Research Centre, Usher Institute, University of Edinburgh, Edinburgh, United Kingdom; 3 Ageing and Health, Usher Institute, University of Edinburgh, Edinburgh, United Kingdom; 4 Forth Valley Royal Hospital, NHS Forth Valley, Larbert, United Kingdom; 5 Population Health Sciences Institute, Newcastle University, Newcastle, United Kingdom; 6 School of Engineering, The University of Edinburgh, , Edinburgh, United Kingdom

**Keywords:** older adult, assistive technology, care, aging in place, barriers, facilitators

## Abstract

**Background:**

Assistive technologies (ATs) are used increasingly in community settings to assist in the care of older adults. Despite a rapid increase in the capabilities and uptake of these technologies, gaps remain in understanding the main barriers to their usage.

**Objective:**

This systematic review investigated the barriers and facilitators to the use of AT in the care of older adults.

**Methods:**

Six electronic databases were searched from January 2011 to March 2024. Primary studies were included if they used qualitative methods reporting findings related to barriers or facilitators to the implementation of AT (eg, ambient and wearable sensors, alarms, telehealth or mobile health [mHealth]) for older adults (from the perspective of either carers or older adults) in community settings. All data were screened independently by two reviewers. Study quality was assessed using the Critical Appraisal Skills Program (CASP). Data from each included study were synthesized using thematic synthesis, before barriers were mapped against the domains of the Technology Acceptance Model (TAM).

**Results:**

Ninety-five studies were included in the review. The number of studies published in the field of barriers to AT use has increased 3-fold post-COVID-19 in comparison to the previous decade. Ten barriers—privacy, cost, insufficient knowledge, fear of misuse, usability, poor functionality, perceived lack of need, stigma, and lack of human interaction—were identified, as well as three facilitators—awareness of health benefits, targeted training, and user-centered design. Persistent barriers relating to all domains of the TAM were identified, with the majority of these relating to the “behavioral intention to use” domain (cost, privacy, stigma, and fear of misuse). The majority of studies had a moderate/high risk of bias.

**Conclusions:**

There remain distinct barriers to sustained usage of AT for the care of older adults, particularly concerning adoption as defined by the TAM. Further studies investigating the acceptability of ATs are needed to increase the understanding of optimization strategies.

## Introduction

Increased life expectancy in many regions around the world has not been matched by improvements in health span (average length of disease-free healthy life) [1]. This has in turn increased the need for health and social care in older populations [2]. Assistive technologies (ATs) could potentially help enable older adults to live independently for longer, which is most people’s preference, and potentially reserve other kinds of more expensive care for those with higher needs. The term ATs covers a range of devices or products designed to support older people to perform activities of daily living such as sensors, wearables, robotics, and information communication technologies (ICT) such as telemedicine [3]. The challenges to in-person delivery of health care experienced during the COVID-19 pandemic led to rapid development and deployment of technology to remotely address gaps in care provision [4].

There are potential benefits of AT for older adults and their caregivers, such as reduced caregiver burden and increased perceived independence [5,6]. However, there is frequently a mismatch between the design, functionality, and usability of AT, on the one hand, and the needs and preferences of end users, on the other hand [7], leading to significant rates of abandonment [8] and barriers to optimal use [7,9-11].

The Technology Acceptance Model (TAM) offers a framework to assess how barriers to AT use may influence sustained adoption [12]. The TAM essentially proposes that an individual’s motivation to adopt new technology can be explained by three factors: perceived ease of use, perceived usefulness, and attitude toward using or behavioral intention to use. Although other comprehensive technology frameworks exist (eg, Unified Theory of Acceptance and Use of Technology 2 [UTAUT2]), the advantage of TAM in relation to others is its relative simplicity, which is often preferred by practitioners and patients over complexity [13].

Systematic reviews of technology adoption in the care of older adults have shown that barriers such as cost, unfamiliarity with technology, perceived lack of need, and poor functionality can all impede the sustained adoption of AT [7,13-17]. However, these reviews either investigated just one technology type [15], only included specific populations with chronic conditions [7,13], or were older than 4 years [16,17]. Given the broad range of available AT, its adoption across diverse older adult care groups, and the pace at which it is developing, this review [18] aimed to synthesize the latest published qualitative literature, informed by the TAM. This then allowed for the identification of barriers and facilitators to implementing a wide range of AT to support paid and unpaid care for older adults.

## Methods

### Protocol Registration

The protocol for this systematic review was prospectively registered on PROSPERO systematic review registry (CRD42021266656) in July 2021 and was reported in accordance with the Preferred Reporting Items for Systematic Review and Meta-Analyses (PRISMA) statement checklist [19] and the Transparency in Reporting the Synthesis of Qualitative Research guidelines [20].

### Search Strategy and Selection Criteria

A comprehensive search strategy was developed in collaboration with a medical librarian prior to execution in 6 electronic databases (Medline, CINAHL, Embase, PsychINFO, Web of Science, and Cochrane Library) from January 2011 to March 5, 2024. Searches were limited by year to capture the most recent evidence and technological advances. Searches included appropriate keywords and medical subject headings. Database searches were supplemented with searches for gray literature in Google Scholar, OpenGray, and MedRXiv preprint server and forward and backward citation searching (to October 2024), including the reference lists of similar systematic reviews found from Google Scholar. The full search strategy used in Medline can be viewed in Table S9 in Multimedia Appendix 1.

### Inclusion and Exclusion Criteria

Primary studies using qualitative methods to understand contextualized personal perspectives of the barriers and facilitators of ATs to support paid and unpaid care for older adults in the community setting were eligible for inclusion. We restricted the search to more recent studies, given rapid changes in AT [21-23]. Barriers and facilitators could be from the perspectives of either older adults or their carers. Studies exclusively containing participants with a single condition were excluded (such as heart failure or diabetes), as a review that only included participants with chronic conditions was published in 2024, which removed the need to synthesize studies in this area [13]. Therefore, this present review only included studies of the general population of older adults receiving care. Given the large volume of studies published in the field of AT for care, this also allowed the review to remain manageable in terms of the number of potentially relevant studies. Similarly, studies exclusively reporting on the use of technology to replace normal care practices during COVID-19 social distancing were also excluded, as these were often temporary and were not planned as a long-term care solution. A detailed description of further inclusion criteria used to select relevant studies is outlined in Textbox 1, while Table 1 describes the definitions applied throughout this review.

Textbox 1. **Inclusion and exclusion criteria**

Inclusion criteria

- Participants were older adults (defined as aged 60 years or older).
- Participants were receiving some form of paid or unpaid care in the community setting (at home receiving domiciliary care or family care, assisted living, care homes, short-term care in the community, eg, rapid response teams to prevent emergency admission, long-term stays in community hospitals).
- The study explores some aspects of either barriers or facilitators of assistive technology usage, or both.
- Technologies explored must be in the context of assisting in the care or independence of older adults (eg, via remote monitoring).

Exclusion criteria

- Studies where participants’ mean age was <60 years.
- Participants are in short-term or acute hospitals.
- A study population made up exclusively of older adults with a specific condition (with the exception of dementia).
- Studies using technology explicitly to replace usual care during social isolation periods of the COVID-19 pandemic.
- Quantitative research.
- Secondary research.
- Study focuses on the general use and acceptability of technologies without exploring barriers or facilitators to actual usage.

**Table 1.** Definitions used for inclusion or exclusion criteria application.

Criteria name	Descriptor
Paid and unpaid care	Any care provided to older adults by health care professionals, third sector organizations (paid/formal care), and friends and family (unpaid, informal care).
Assistive technologies (AT)	Any devices, products, systems, and services that were installed or utilized within a participant’s living environment, community-based care environment, or on their person, with the primary aim to assist in activities of daily living or with the process of care provision through remote monitoring or data generation for care provision.
Facilitators	Factors that increase, improve, promote, facilitate, or enable the use of AT.
Barriers	Factors that prevent, obstruct, or decrease the use of AT.

### Screening and Data Extraction

Consistent with the Joanna Briggs Institute Manual for Qualitative Evidence Synthesis [24], title and abstract and full-text screening were undertaken by two independent reviewers using Covidence® systematic review software, with conflicts discussed and resolved by consensus (SM screened all studies, with second screening by either BG, AOD, HF, SDS, SWM, BB, AE, KM, or CP). Data extraction was undertaken using a predeveloped data extraction template covering study identifiers, methods, population, technology, and facilitators and barriers. One reviewer extracted all studies, with a second reviewer checking extraction for accuracy, with any discrepancies discussed and resolved by consensus.

### Quality Appraisal

Studies were assessed for quality using the Critical Appraisal Skills Program (CASP) tool for qualitative studies [25], extracting methodological issues that could be sources of bias, rather than assigning scores of quality.

### Thematic Synthesis

We adopted the principles of framework analysis to conduct a deductive thematic synthesis [26]. Two researchers with qualitative experience (SM and AOD) independently coded facilitators and barriers from two primary studies to develop a working analytical framework, which was then tested on a further study. After modifications, the remaining studies were coded by the lead author. During the final stage of data interpretation, facilitator and barrier themes were developed by the lead author by looking at the data set as a whole and through discussions with the wider team, in addition to categorizing studies by participant type (ie, carer, older adult, or both). Illustrative quotes relating to each theme are presented in Table S6 in Multimedia Appendix 1 to provide context. Following thematic synthesis, barriers were mapped onto the domains of the TAM (by SM) to help identify aspects of technology adoption which may present greater need for support, or where particular interventions can facilitate usage. Specifically, identified barriers were aligned with the stage of the TAM at which they were determined to present themselves within the literature. For example, cost is a barrier that mainly prevented participants from uptaking AT in the first place; therefore, this barrier would be mapped to the “behavioral intent to use” TAM domain. While a multitude of technology acceptance frameworks have been developed [27], the TAM was selected as the most appropriate framework for this review for two reasons. First, the TAM was developed using two behavioral theories which directly influence adoption behaviors; namely, theory of reasoned action [28] and theory of planned behavior [29]. Second, the TAM is a simple model consisting of five easily interpreted domains, rendering it a useful framework for a wider audience to comprehend or apply to their own work, thus potentially increasing transferability of the review findings.

Additionally, the proportion of barriers within each TAM domain for papers classified as pre-COVID (ie, primary studies indicate data collection was conducted prior to January 2020) or post-COVID (ie, primary studies indicate in methods that data collection was conducted post January 2020) was calculated. Results were summarized to observe both the volume of papers published in these two periods and any proportional change in TAM domains (represented by different colors) covered by identified barriers.

## Results

### Summary of Included Studies

**Figure 1.**
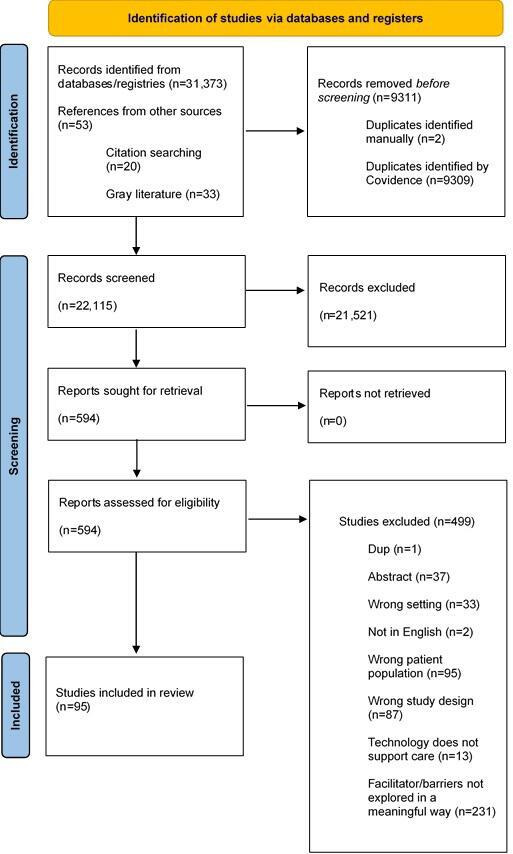
PRISMA flow diagram of review. “Reports” indicates a full text document for screening. “Studies” indicates individual investigations. PRISMA: Preferred Reporting Items for Systematic Review and Meta-Analyses.

Ninety-five studies met inclusion criteria (Figure 1). There was considerable study-to-study variation in both the type of technologies reviewed and the level of detail reported regarding the technologies. Studies related to AT facilitated remote monitoring. The most commonly investigated technologies were telehealth or mHealth systems, generic information communication technologies such as smartphones, and other communication devices such as tablets (n=44). Smart home technologies such as ambient sensors were the second most common technologies (n=23), followed by wearables such as sensors and alarms (n=13). The remaining studies investigated workforce support tools such as electronic health records or decision support tools (n=8) and robots (n=10); four studies reported on findings for more than one AT type. Further details on each included study can be viewed in Tables S1-S5 in Multimedia Appendix 1.

### Study Quality

Overall, studies included in this review effectively specified their research question—target population group—and described their findings adequately in relation to the aims of the study. Recruitment strategies were often not reported in sufficient detail. Additionally, in general, studies did not adequately report steps taken to ensure rigor of findings, with only five studies adequately considering any potential influences the researcher(s) may have had over participants (eg, whether the researcher is known to the participants, is an external evaluator, or a member of the intervention implementation team), in turn meeting all criteria of CASP [30-34]. Some studies provided limited descriptions of the types of technologies tested in the studies, which in turn made defining specific barriers between technologies challenging. The majority of studies were small scale and of a moderate to high risk of bias overall. Table S7 in Multimedia Appendix 1 provides full CASP scores for all included studies.

### Barriers to AT Use

Three of the 10 themes were related to technology design (intrusive/discomfort, poor functionality, and usability; see Table 2). The most common barrier across all technology types was usability, and lack of knowledge was also consistently identified (eg, lack of experience with technology, unfamiliarity with digital interfaces, etc). Stigma showed considerable variation, with no barriers related to stigma identified for telehealth or mHealth but 30% (n=4) of studies assessing wearables reporting stigma as a barrier. In particular, stigma was more common in wearable technologies and alarm systems, which were perceived to draw attention to users. Indeed, certain barriers appear to be unique to a specific AT. For example, discomfort was cited as a barrier to use in 36% (n=5) of wearable studies but not across any of the other AT groups. Similarly, lack of human interaction was more common in smart home technology, robots, and telehealth or mHealth but not cited for wearables or workforce support. A narrative summary of each identified barrier and facilitator is provided below, with supporting quotes available in Table S6 in Multimedia Appendix 1.

**Table 2.** Overarching barrier themes with descriptions.

Barrier and facilitator theme names	Descriptor
Barriers	
Privacy	Any reference to perceptions of the AT invading personal privacy, such as data breaches, being watched, or having access to personal information
Cost	Any reference to the financial implication of using AT, such as the cost of the equipment, its upkeep, or subscription cost.
Usability	Any reference to how easy the AT is to use. For example, if buttons are hard to press or screens are small, making legibility difficult
Poor functionality	Distinct from usability. Any perception that the AT does not work as it is intended to, reducing confidence in its effectiveness, for example, false alarms, poor battery life
Stigma	Any perception that using AT draws negative attention to the user such as pity or perceptions of frailty
Intrusive/discomfort	Distinct from privacy, AT that is uncomfortable to wear or creates obstacles in home
Lack of need	Perception that the AT is not required for care or that the participants do not feel they require such devices for their health
Lack of knowledge	Perception that the participant does not know enough about the correct way to use the AT to use it effectively
Fear of misuse	Reluctant to use AT for fear of breaking it due to incorrect use or compromising safety/privacy through incorrect use
Lack of human interaction	Perceptions that AT is replacing human carers to the detriment of older adults, for example, reduced carer visits due to smart home monitoring, reducing social interactions
Facilitators	
Tailored training	Any form of structured training/education in the use of AT
Viewed positively by trusted relations	Carers, medical professionals, or family members encourage the use of the AT and advocate for it
Support	Any informal help from carers, friends, or family members to be more proficient at using AT

a AT: assistive technology.

### Identified Barriers by Study Participant Group

**Figure 2.**
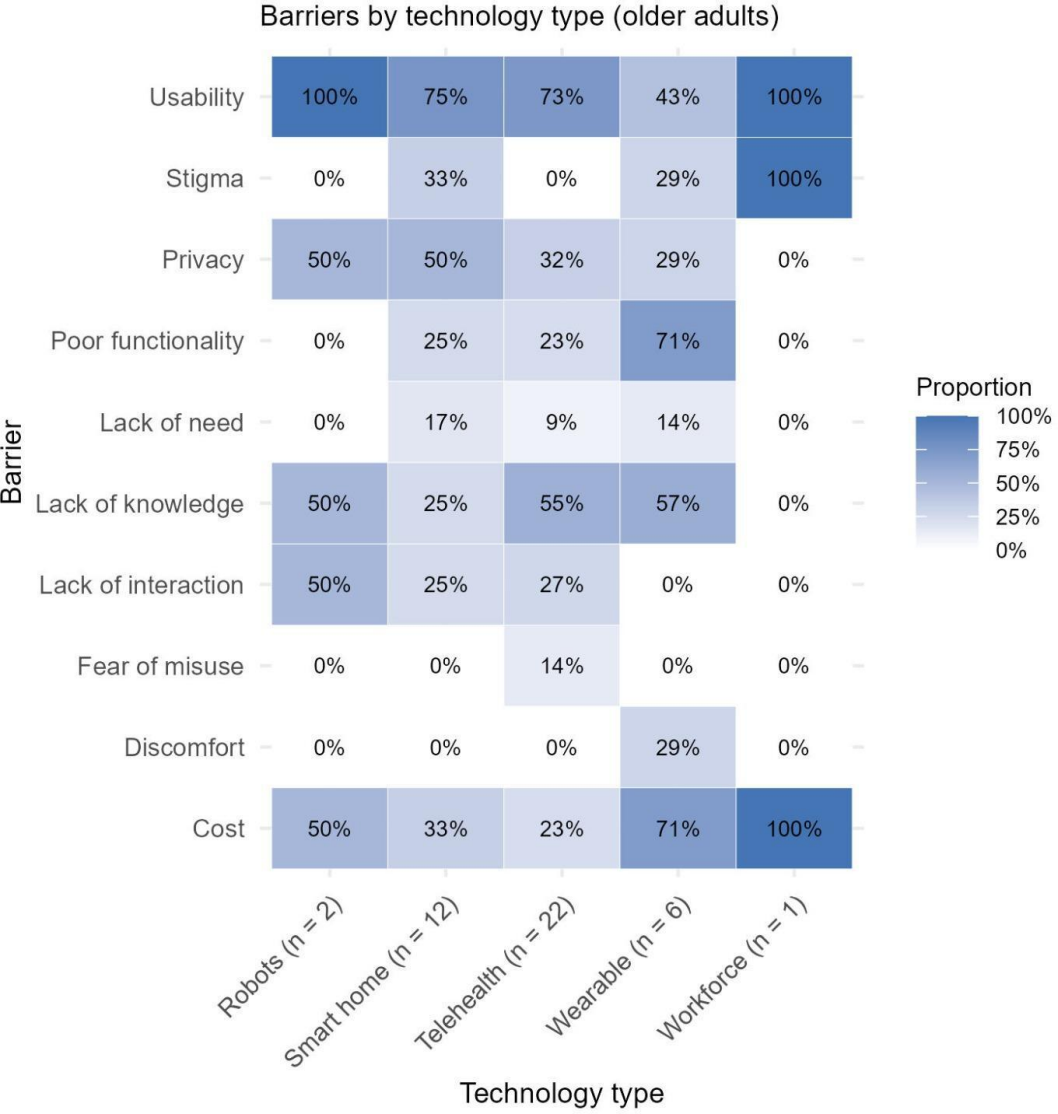
Matrix plot of proportion of barriers identified across technology types for studies including older adults only.

The majority of studies exclusively included older adults as the research participants, with no carer perspectives included (n=43). Conversely, a minority of studies exclusively recruited carers (n=17), with the remaining studies (n=36) including both older adults and carers in the study sample. Of these combined studies, presentation of distinct findings by each group was limited, making separation of barriers or facilitators experienced by older adults versus carers challenging. Figure 2 present the proportion of barriers for each technology type across studies of older adults, carers, and combined samples separately. When identified barriers between the carer group and older adult group are considered, there are clear differences in the proportional distribution of some barriers. For example, no barriers related to stigma were identified for any technology among carers, while this was a significant barrier for older adults, particularly regarding wearables and smart home technologies. Lack of human interaction also appeared to be more of an issue across technology types for older adults than for carers. Conversely, carers generally identified lack of knowledge as a barrier across all technologies to a greater extent than older adults.

**Figure 3.**
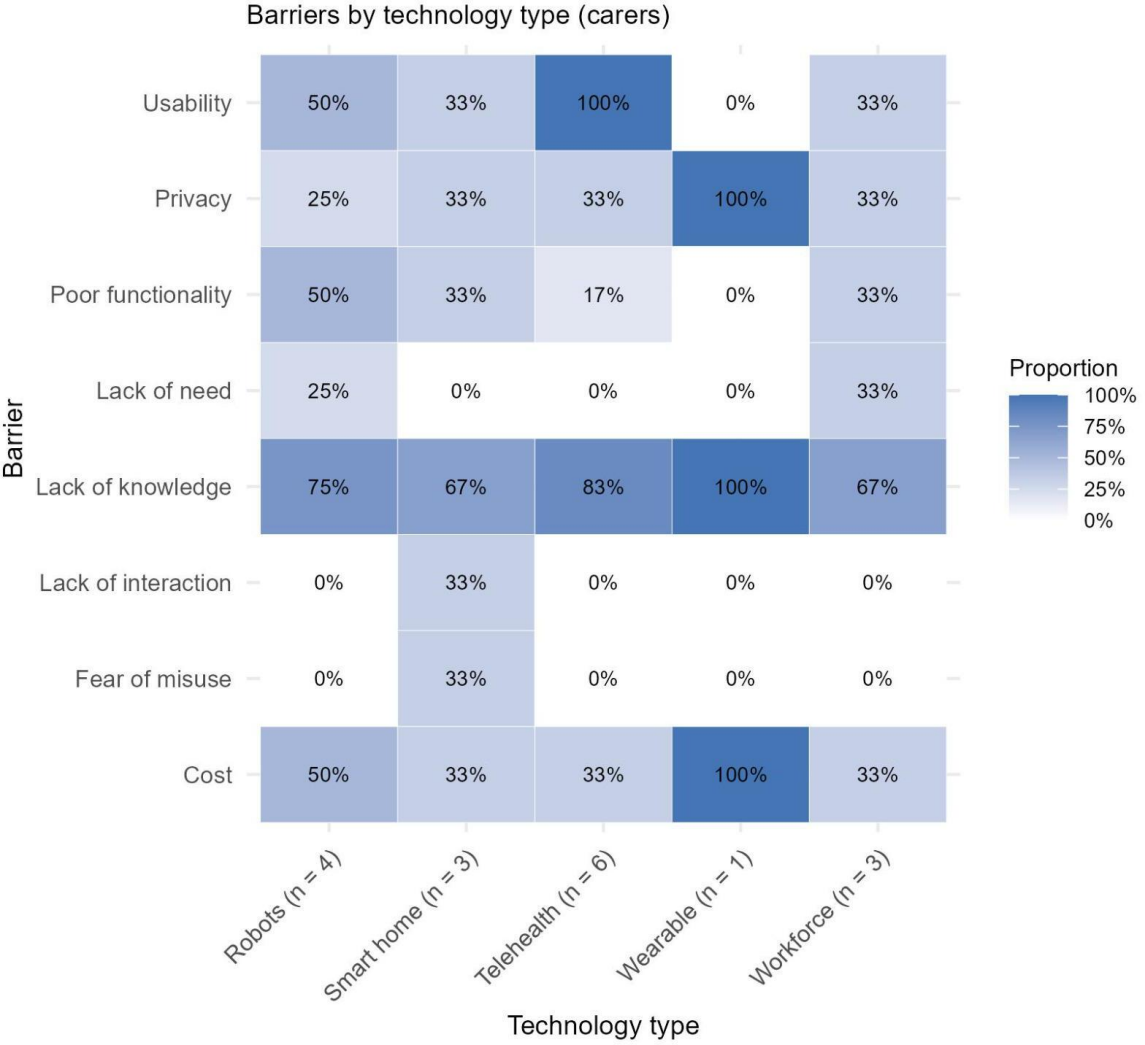
Matrix plot of proportion of barriers identified across technology types for studies including carers only.

**Figure 4.**
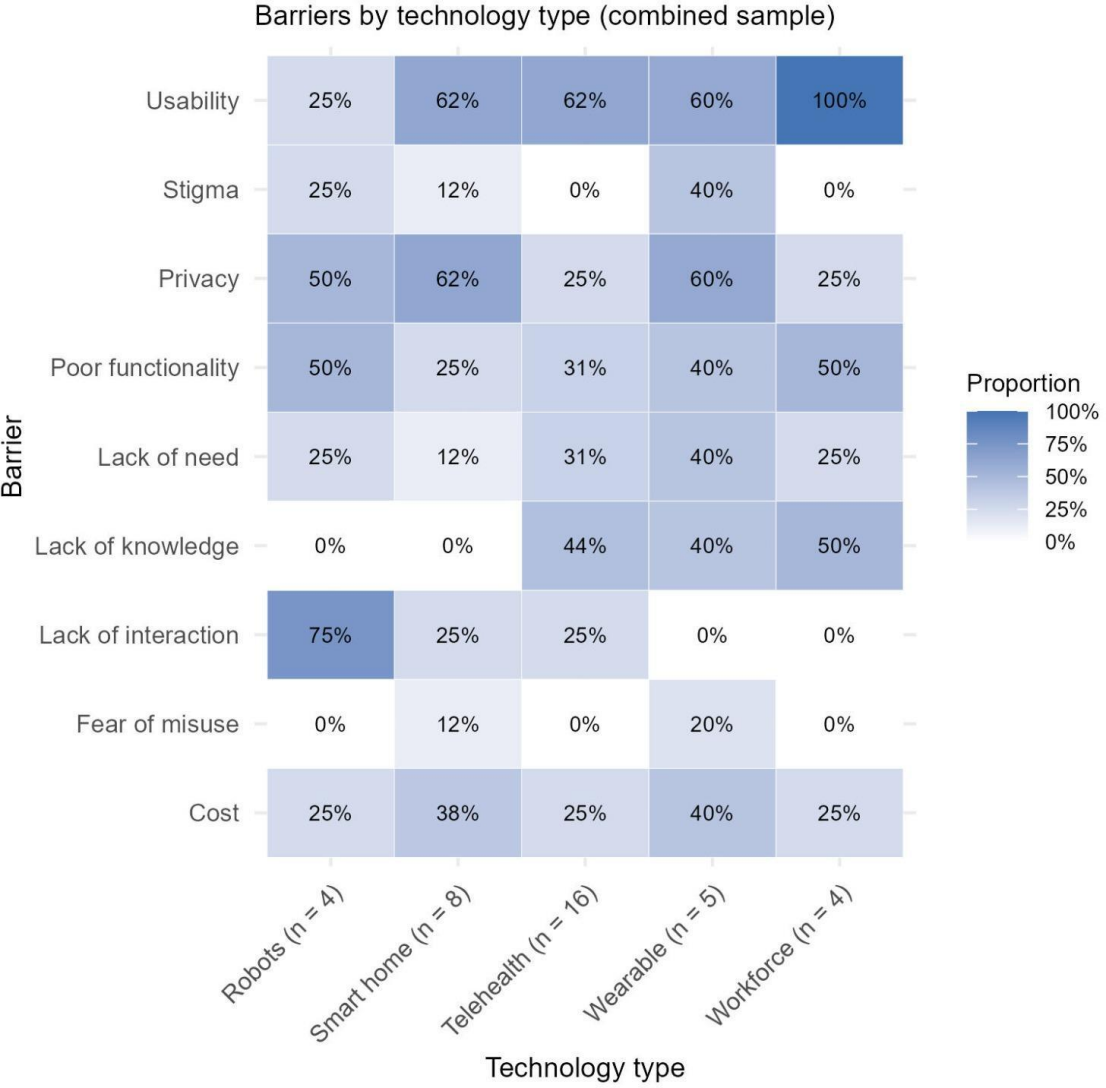
Matrix plot of proportion of barriers identified across technology types for studies including carers only.

### Technology Design

#### Usability

Usability was the most persistent barrier across all AT types (Figures2^4^) [5,30,31,34-87]. Complex interfaces, intricate buttons/controls, and small text/letters coupled with physical and cognitive limitations were all cited as barriers to effective use of these technologies.

#### Poor functionality

Poor functionality was a persistent barrier across all AT types [33,35,37-39,41,46,55,60,70,71,80,81,85,88-102]; this pertained to the perception that AT did not actually address the issue it was designed to assist with. This was particularly true where the technology failed or did not work as intended (eg, battery life of sensors was not fit for purpose, inaccurate alarms sounding when no adverse event had taken place, or software that crashed).

#### Discomfort

Some wearable devices (but not other AT types) were also viewed as uncomfortable to wear, which discouraged usage. Four studies [61,81,97] highlighted that participants did not like wearing the device as it felt uncomfortable and “in the way.”

### Other Barriers Related to Particular Technologies

#### Cost

Cost was a major barrier identified across AT types, with 28 studies citing concerns with the financial implications of installing or using AT [5,32-34,38,43,45,47,49,50,53,58,59,70,75,77,82,83,94,96,99,100,103-107]. There was a perception that there would be ongoing costs associated with owning these technologies.

One study of falls prevention technology highlighted that financial cost was only identified as a barrier for participants in their sample that were considered low income but not for more affluent participants [92], highlighting the potential for AT to widen health inequalities if the barriers imposed by cost are not considered from the outset.

Out of all the barriers identified in this review, cost was one for which there could be potential contextual implications relating to geographical location. Specifically, as the United States is the only high-income country without universal health care, it could be theorized that cost would be a more pronounced barrier among US-based studies. However, of the 27 US-based studies included in this review, only 9 (33%) identified cost as a barrier, which does not considerably differ from the 24 out of 69 (34%) non US–based studies that found cost to be a barrier.

#### Lack of knowledge and fear of misuse

A lack of knowledge or general fear and anxiety about using technology incorrectly or breaking devices was common. This barrier was particularly evident in studies focused on technology that required participants to interact with software, such as smartphones, wearable devices, or computers [51,66,74,85,97,98,108,109]. In contrast, lack of knowledge was distinctly lower proportionally for smart home technologies, which tend to be more passive in their function (Figures2^4^).

This lack of knowledge and fear of incorrect use often led to feelings of anxiety among older adults that they were unknowingly using the technology wrong, which could lead to negative consequences (eg, inaccurate information being communicated to their providers regarding their health) [98].

#### Perceived lack of need

Perceived lack of need was a persistent barrier to AT usage, particularly for technologies designed to assist older adults to live independently (such as robots or fall prevention systems). This relates to individual self-perception that they were not yet of the age or physical condition to require such assistance, even when it was recommended by health care professionals or family members [44,48,57,62,66,78,92,94,98,104,106,110-112]. One study of older adults’ usage of technology to assess frailty in the home demonstrated that although participants acknowledged that such technology could be useful in older age, they did not think that they personally needed such AT to live independently at this point in time, a sentiment shared by participants in other studies [88]. Similarly, some of the technologies reported in this review were perceived by participants (both older adults and caregivers) as simply not useful [88,109].

### Concern About Adverse Consequences

#### Lack of interaction

Lack of interaction was a commonly identified barrier in studies relating to smart home tech, telehealth or mHealth, and robots, in the sense of ATs leading to a decrease in human interaction and opportunities to have informal discussions with health care providers [5,31,35,45,47,52,56,62,84,103,104,113-115]. For some individuals, this represented one of their only opportunities to get out of their home and meet another person (eg, a health care provider), or for someone to visit them (eg, a carer).

#### Privacy

Thirty-eight studies identified privacy concerns as a barrier to AT usage [5,30-32,34,36,44,45,47,50,56,58,63,67-70,72,75,77,85,89,96,99,100,105,111,112,115-117], either due to perceived intrusion on personal data or potential invasion of personal privacy by smart home technologies. Both older adults and carers in a number of studies perceived the installation of technologies such as ambient sensors or cameras as an invasion of personal space and privacy, which could make them feel uneasy in their own home.

Older adults also expressed concern that health professionals, insurance companies, or members of their family would be receiving private or delicate information regarding their health status, movements, or activities, which was perceived as an invasion of privacy.

#### Stigmatization

Stigmatization or general awareness of what others would think of them as a result of AT use was also a persistent barrier among older adults identified in ten studies [53,59,70,79,89,92,103] . This was evident in a study of ATs for older adults with age-related vision loss.

Stigma was most prevalent as a barrier to use of wearable technologies, while no studies relating to telehealth or mHealth cited stigma as a barrier. This is likely due to the attention some wearables can draw to the individual due to audible alarms or the visual design of the AT [118].

### Facilitators to AT Use

#### Overview

As is common in research on barriers and facilitators in health care, many of the facilitators identified in this review directly reflect the removal of the barriers described above. For example, affordability, privacy measures, and ease of use were all identified as facilitators to AT use. Below we explicitly describe three identified facilitators that are distinct from simple removal of barriers.

#### Awareness of Benefits

Certain ATs were highlighted as allowing older adults to learn more about their own health behaviors and daily activities, which was viewed as a facilitator (particularly for wearables) [119,120]. If technology was perceived to increase safety and independence, participants were more likely to state that they would be willing to use it in a number of studies [40,119,120]. For example, across multiple studies of ambient sensors, participants highlighted that the AT offered real benefits by increasing safety and reducing caregiver burden.

#### Targeted Training

One of the most consistent facilitators to AT usage cited in the included studies was the use of training for older adults on how to properly use the technologies they were to be given [54]. This included strategies such as preimplementation of training materials on AT use to familiarize older adults with the technology [39], and one-to-one or group assistance with usage during initial implementation [53].

#### User-centered design

A user-centered design was viewed as a facilitator of more user-friendly technology development in 23 studies. Participants in a number of studies cited efforts to involve users in the design process as a benefit to usability. For example, Young and colleagues [87] employed an iterative design process (involving formal interviews and journey mapping) to develop a digital home cognitive screening tool. They found this person-centered design approach to be an asset in developing an AT that was acceptable across different user groups.

### Barriers and Facilitators in Relation to the TAM

**Figure 5.**
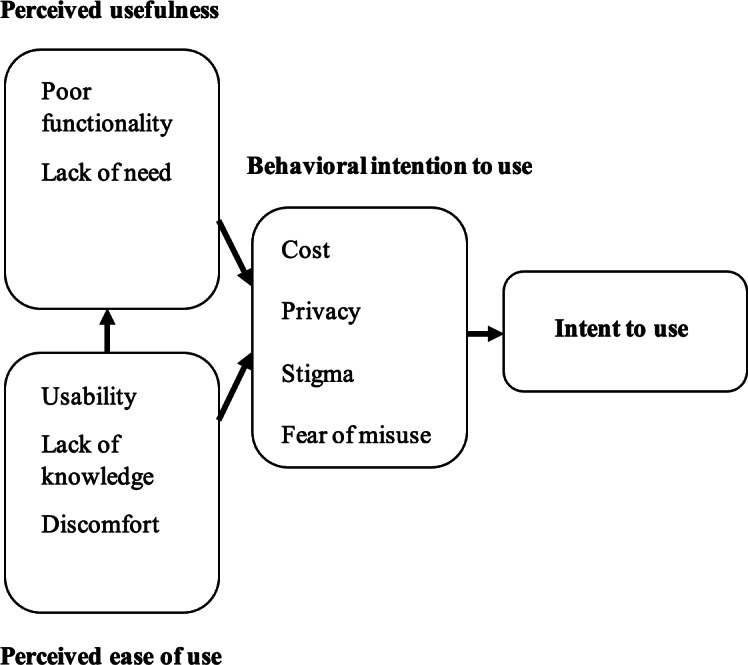
Identified barriers to assistive technology use mapped against the technology acceptance model.

Figure 5 illustrates how all 10 of the identified barriers from the primary studies map onto the TAM, highlighting the points at which specific barriers present problems in sustained use of AT for care. While the majority of these aligned with the “behavioral intention to use” domain of TAM (cost, privacy, stigma, fear of misuse, and lack of human interaction), barriers such as poor functionality and lack of need impacted on “perceived usefulness,” and three barriers (discomfort, usability, and lack of knowledge) mapped onto the “perceived ease of use” domain. Likewise, ATs that were found not to work as intended, or where no explicit care need was perceived to have been addressed, corresponded with perceived usefulness domain. All identified barriers were able to be mapped against a domain of the TAM (Figure 5).

### Barriers to AT by TAM Domain Pre- Versus Post-COVID

**Figure 6.**
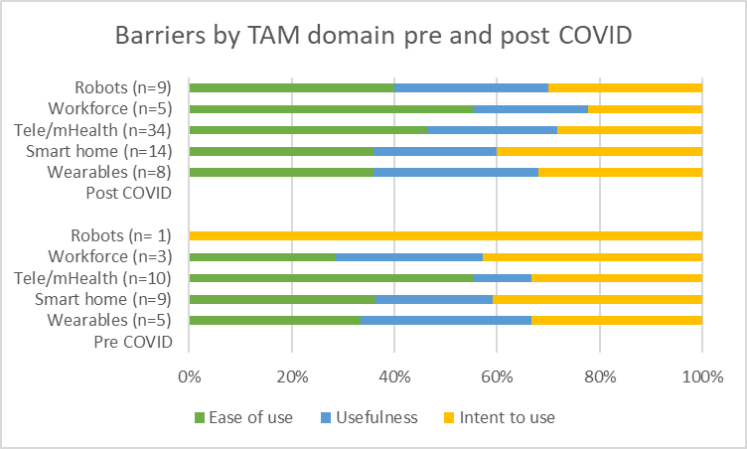
Proportions of identified barriers by TAM domain for studies published pre (2011‐2020) and post COVID (2021‐2024). TAM: Technology Acceptance Model.

Figure 6 illustrates the potential influence that the COVID-19 pandemic may have had on both the quantity of studies assessing AT for care and their acceptance by users in relation to the domains of the TAM.

The type of barriers and their proportions in relation to the TAM is generally similar between pre- and post-COVID. However, for robots, studies identified further barriers in ease of use, usefulness, and intent to use. In telehealth or mHealth, there was a proportional increase in usefulness barriers, and for workforce support tools, there was a proportional increase in usability barriers. There was no marked change in barriers relating to intention to use AT in any technology type.

## Discussion

### Principal Findings

This systematic review identified 10 barriers related to the use of AT for the care of older adults in community settings. These mapped onto various domains across the TAM, highlighting the point at which such barriers may negatively influence adoption. The findings of this review are reinforced by previous reviews on this topic [13,15,16]. For example, privacy of AT was a consistent barrier, reflecting the findings of previous reviews [15,17]. Specifically, Yusif et al [17] identified privacy to be the most prevalent barrier identified in their review, being cited within 34% (n=15) of the 44 included studies. Similar findings were observed for usability, both for our review and existing reviews [14,17]. Also, in line with the findings of our review, Kruse et al’s [14] review of 57 telehealth and mHealth acceptability studies found both technical illiteracy and a lack of desire to be two of the most prevalent barriers identified. This reflects findings in our review whereby perceived lack of need and lack of knowledge were persistent barriers identified across all AT, including studies of telehealth and mHealth. Stigma was also cited as another barrier to AT adoption across several reviews [14,15,17]. This appears to be more prevalent in ATs that are perceived to draw attention to the user, such as body-worn sensors or alarm systems [118]. Wang et al’s [16] mixed methods review of long-distance care technologies including 41 studies highlighted the importance of considering personal factors which may mediate adoption of AT among older adults. Specifically, their review highlighted that factors such as individual personality and cohabiting circumstances can influence AT adoption, just as frequently as technology factors such as poor functionality and usability. These personal factors are often more challenging to capture in primary studies, highlighting the need for more robust study designs such as controlled studies with representative participant groups. Additionally, Vassli and Farshchian’s [15] systematic review of qualitative studies investigating ICT adoption in older adults mapped identified themes to the UTAUT2. Barriers identified largely mirrored those identified in our review (cost, functionality, lack of knowledge, lack of human interaction, discomfort, and stigma). Mapping the UTAUT2 provided further nuance to these findings, highlighting where specific barriers may influence adoption in relation to each domain. For example, obtrusiveness or discomfort was theorized to negatively impact effort expectancy, thus in turn reducing behavioral intention to adopt intrusive ICT, while stigma could be classified as a barrier to adoption within the social influence domain of the UTAUT2 [15].

To our knowledge, this is the first review to also assess the barriers of a comprehensive range of AT types in conjunction with stages of the TAM. This exercise allowed for the identification of the barriers to AT use in relation to TAM domains. In conducting this exercise, it was important to acknowledge that the COVID-19 pandemic increased the volume of research output in this field exponentially and is likely to have expedited the pace at which a number of technologies have progressed from state of the art to well established and commonly used in the care of older adults [18]. What is less clear, however, is whether these advances in widespread use of ATs have also coincided with progress in addressing the persistent barriers, which prevent older adults from optimally engaging with care technologies. This review has demonstrated an almost 3-fold increase in the volume of studies in this area, within a third of the timeframe post-COVID-19. It is important to note, however, that it was not possible to identify the mechanisms and drivers behind the increase in research output post-COVID-19. While the pandemic and the necessity to adhere to social distancing played a role, the natural increase in pace and progression of technological advances was also a factor.

Using the TAM domains to map identified barriers, our analysis demonstrates persistent challenges in addressing barriers, which affect older adults’ behavioral intention to use technologies. Barriers relating to ease of use and perceived usefulness are largely similar in proportion for both pre and post COVID-19, with the exception of robots, for which a large increase is observed mainly due to more studies now being published versus the one study that existed pre-COVID. This indicates that despite the increased rate of publication in the field, barriers remain the same as those which impeded AT use before the pandemic.

This is unsurprising given the known complexities involved in influencing behavior change in clinical interventions [121]. In older studies of AT adoption, barriers relating to ease of use and perceived usefulness tended to be easier to rectify with alterations to AT hardware or software (albeit with unique challenges related to use by older adults, who often have additional needs such as frailty or vision loss that can require products to need further optimization) [122]. Conversely, addressing barriers such as perceived stigma, fear of misuse, or a lack of human interaction requires more than adaptations to the AT itself and will likely require multicomponent strategies to address the cultural and behavioral issues around the adoption of such technologies [16]. While both primary research and systematic reviews such as the present study can help identify barriers to AT use, adopting strategies to overcome such barriers will require careful planning from all stakeholders. Implementation science frameworks offer researchers and practitioners a viable avenue to achieve this [123]. One such framework that has been successfully applied to technological interventions is the Consolidated Framework for Implementation Research (CFIR) [124,125]. Briefly, CFIR consists of five domains (intervention characteristics, outer setting, inner setting, individual characteristics, and implementation processes). These domains contain further constructs upon which identified barriers of an intervention or ICT can be mapped in order to determine relevant implementation strategies [126]. If used in conjunction with the TAM, these implementation strategies can be further optimized to suit the stages at which barriers occur in the adoption process, as recent studies have achieved. For example, Gallant et al [127] conducted a mixed methods preimplementation study whereby the CFIR was used in conjunction with UTAUT to inform the implementation of automated pain behavior monitoring technology in a health care setting, leading to increased understanding of behavioral intentions to use technology and refinements to the UTAUT, which may help in making the framework more widely applicable to practice. Therefore, the results of this review, which has mapped commonly identified barriers in AT adoption to the TAM, can help researchers to expedite this process when adopting frameworks such as CFIR.

Strengths of this review include the comprehensive search strategy and the consideration of the potential influence of the COVID-19 pandemic to understand how this has influenced technology acceptance. There are a number of limitations that should be considered when interpreting the findings. For example, following the risk of bias assessment using CASP, a large portion of the included studies was deemed to be of moderate to high risk of bias, limiting the trustworthiness of the findings reported. Specifically, studies generally did not adequately address the relationship between the interviewer and the participants. This observation is not limited to this current review. A review of qualitative study methodologies found that none of the 19 included articles adequately described reflexive practice in data collection/analysis [128]. It was noted that most of the included studies appeared to be stand-alone, small-scale feasibility studies. While such studies are an important step in the refinement of ATs, their formative status can limit their generalizability when attempting to use such findings to inform practice at scale [129]. Additionally, previous reviews of the literature have highlighted that participants in studies of technology acceptance tend to be higher than average socioeconomic or educational status and at the younger end of older age [130]. Similar issues likely apply to the studies included in this review (Tables S1-S5 in Multimedia Appendix 1), which could limit generalizability. Finally, we applied a pragmatic approach to data synthesis, using double coding of a proportion of studies to develop a coding framework, before single coding was applied to the remaining studies as is common practice in reviews of a similar nature [131]. Consequently, there is an increased risk of misclassification bias of identified themes due to this. However, given the barriers typically identified in AT use among older adults are fairly distinct and straightforward to define, the true impact of this methodological limitation is likely minimal.

### Conclusions

The scope and scale of ATs offering support to older adults and their carers continues to expand. However, ATs commonly are not optimized for use in this context, which could be addressed by better codesign or user-centered design (TAM domains of usefulness and ease of use). Other important barriers relate to the TAM domain of behavioral intent to use ATs, which requires a different set of interventions. Future research should take an implementation science approach by identifying solutions to the barriers to AT use, while conducting more rigorous evaluations of optimized ATs accompanied by robust process evaluations to help identify the impact of any persistent barriers on adoption and other outcomes of interest.

## Supplementary material

10.2196/73917 Multimedia Appendix 1 Full study description tables, full text exclusions with reasons, CASP quality assessment scoring, and MEDLINE search strategy.

10.2196/73917 Checklist 1 PRISMA checklist.

## Abbreviations

AT: assistive technologies
CASP: Critical Appraisal Skills Program
CFIR: Consolidated Framework for Implementation Research
PRISMA: Preferred Reporting Items for Systematic Review and Meta-Analyses
TAM: Technology Acceptance Model
UTAUT: Unified Theory of Acceptance and Use of Technology
