# Supplementary material for: Patient and Carer-Related Facilitators and Barriers to the Adoption of Assistive Technologies for the Care of Older Adults: Systematic Review
Source: JMIR Aging. 2025 Nov 27;8:e73917. doi: 10.2196/73917 (PMC12661617; doi:10.2196/73917)
Supplement: Multimedia Appendix 1 [file aging-v8-e73917-s001.docx]

# **Supplementary materials for Barriers and facilitators to implementing assistive technologies to support paid and unpaid care for older adults: a Technology Acceptance Model-informed systematic review.**

**Contents:**

| **File name** | **Page number** |
| --- | --- |
| Supplementary Table S1: Studies of wearable devices | 2 |
| Supplementary Table S2: Studies of smart home technologies | 4 |
| Supplementary Table S3: Studies of Telehealth, mHealth, and Communication Tools | 6 |
| Supplementary Table S4: Studies of Workforce Support Tools | 10 |
| Supplementary Table S5: Studies of Robots | 11 |
| Supplementary Table S6: Supporting quotes for qualitative findings | 12 |
| Supplementary Table S7: Quality assessment scoring | 16 |
| Supplementary Table S8: Excluded full text articles with reasons | 20 |
| Supplementary Table S9: MEDLINE search strategy | 75 |

**Supplementary Table S1: Studies of wearable devices**

| **Author & Year** | **Country** | **Main Focus** | **Participants** | **Technologies** | **Barriers** | **Facilitators** |
| --- | --- | --- | --- | --- | --- | --- |
| Chaudhuri et al., 2017 | USA | Experiences with a novel fall detection device | 27 older adults (55% women, aged 62+), recruited from assisted and independent living communities | Wearable fall detection device (GPS, accelerometer, magnetometer, communication) | Poor functionality, lack of need  Stigma, cost | Awareness of benefits |
| Hall et al., 2019 | UK | Older adults' perceptions of hip protectors | 147 older adults (55-96 years) and 10 care staff, recruited from community and housing facilities. | Wearable hip protectors with sensors | Stigma  Poor functionality  Perceived lack of need cost | Awareness of benefits |
| Kononova et al., 2019 | USA | Motivators and barriers for activity trackers | 48 participants (65-94 years, 73% women), recruited through senior centers and local ads. | Wearable activity trackers (Fitbit, Garmin Vivofit 2) | Poor functionality  Intrusive  Cost  Lack of knowledge | Awareness of benefits |
| Parker et al., 2013 | USA | Older adults’ use of mHealth for pain management | 41 participants (aged ≥60, mean age 76.2, 78% female; 56% White, 34% African American) | wearable pain trackers | Poor functionality, cost, privacy, lack of knowledge | Tailored training, |
| Thilo et al., 2017 | Switzerland | Needs and preferences for a wearable fall detection device | 22 participants (aged 75-89, 82% female), recruited via senior associations and healthcare institutions | Mock-up wearable fall detection device paired with a smartphone | Usability, intrusive/discomfort, poor functionality | Tailored training |
| Kim et al., 2021 | Canada | Perceptions of clinicians and older adults regarding PGHD for chronic conditions | 9 participants (4 clinicians, 5 older adults, mean age 81, 80% female) | Wearable devices for collecting PGHD | privacy, lack of knowledge, usability | Reduced carer burden, awareness of benefits |
| Gomez et al., 2021 | UK | Barriers and facilitators to smartphone-connected hearing aids | 8 hearing aid users (mean age 71.75, range 65-81, digitally literate) | Smartphone-connected hearing aids | Lack of knowledge, poor functionality | Tailored staff training |
| Gordon et al., 2022 | Australia | Evaluating the effectiveness of a co-designed technology package on safety perceptions and well-being | 30 participants (mean age = 79 years; 73% female, 83% living alone; 50% had a low fall risk) | Fall detection monitors, digital assistants, memory compensatory devices | Cost, lack of knowledge, usability, stigma | Tailored training, user-centred design |
| Neubauer et al., 2021 | Canada, UK, Ireland | Adoption and usability factors of locator devices for persons with dementia | 21 participants (service providers, tech developers, care partners, PLWD with mild cognitive impairments) | Locator devices (GPS tracking, geofencing, two-way communication) | Cost, usability, privacy concerns, stigma, poor functionality | User-centred design |
| Dequanter et al., 2022 | Belgium, Canada | Perspectives of professional caregivers on assistive technologies for older adults with mild cognitive decline | 14 participants in two focus groups (aged 23-60, mostly female, multidisciplinary caregivers including occupational therapists and nurses) | Geo-tracking systems, fall detection systems, calendar clocks, pill dispensers | Lack of knowledge, privacy, cost | / |
| Gris et al., 2023 | Italy | Co-designing DemiCare to support caregivers of PwD | 2 PwD (aged 73 and 85) with mild/moderate dementia; 3 ICs (aged 51-69); 7 HPs (psychologists, biomedical engineer, healthcare worker) | DemiCare system (smart soles, smartwatches, mobile app) | Perceived lack of need, privacy, lack of knowledge | User-centred design |
| Garcia Reyes et al., 2023 | Australia | Older adults' experiences with health self-management technologies | 22 older adults (64% female, aged 65-87, mean age 73) | Telehealth, fitness trackers, medical devices | Usability, privacy, cost | User-centred design |
| Mishra et al., 2023 | USA | Acceptability of Care4AD platform for dementia care coordination | 35 stakeholders: 10 PwD (mean age: 73.4 years, 50% female), 14 caregivers (mean age: 68, 75% female), 11 dementia care experts | Wearable sensors, tags for ADL monitoring | Usability, fear of misuse, | Awareness of benefits, reduced caregiver burden |

**Supplementary Table S2: Studies of smart home technologies**

| **Author & Year** | **Country** | **Main Focus** | **Participants** | **Technologies** | **Barriers** | **Facilitators** |
| --- | --- | --- | --- | --- | --- | --- |
| Bian et al., 2021 | Canada | Perspectives on technologies for assessing frailty | 15 older adults (60% women, aged 65+), recruited from ongoing study and local hospitals | Chair/bed sensors, motion sensors, door sensors, smart speakers, fridge door sensors | Privacy, usability, stigma | User-centred design |
| Cohen et al., 2016 | Switzerland | Acceptability of intelligent wireless sensor systems (IWSS) | 34 older adults (aged 65+, informal caregivers) | IWSS (fall detection, health monitoring, integrated sensors, alarms) | Usability, poor functionality, lack of need | Reduced caregiver burden |
| Davenport et al., 2012 | USA | Decisions regarding smart technology | 11 older adults (majority women, aged 65+), with mobility impairments | Smart walkers, fall detection systems, sensors | Cost, stigma, reduced human interaction, lack of need | Awareness of benefits, reduced caregiver burden |
| Elers et al., 2018 | New Zealand | Technology for aging in place | 10 older adults (aged 70+), supported by 31 informal network members | Home monitoring devices, smart home systems | Cost, privacy, usability, reduced human interaction | / |
| Gottwald et al., 2016 | Finland | Usability of public participation GIS tools | 20 older adults (59-77 years, 80% women), recruited from the Kamppi Service Centre. | Public participation GIS (PPGIS) tools | Usability | Tailored training |
| Orellano-Colón et al., 2015 | Puerto Rico | Barriers to assistive tech (AT) for functional limitations | 60 Hispanic older adults (70-97 years, 67% women, low-income). | Mobility and hygiene aids, medication management tools | Lack of knowledge, cost, stigma. | Tailored training |
| Pol et al., 2016 | Netherlands | Perceptions of sensor systems for independence and safety | 11 older adults (aged 68-94, 7 women, living alone in the community or senior residences) | Sensor monitoring systems (motion, door/cabinet, toilet sensors) | Privacy | Awareness of benefits |
| Wang et al., 2019 | USA | Older adults' perspectives on smart tech for aging in place | 31 participants (aged 67-94, mean 80, 64.5% women) residents of a senior housing community | Smart technologies including IoT, AAL, AI systems | Usability, privacy, poor functionality, lack of knowledge | Awareness of benefits, user-centred design |
| Dermody et al., 2023 | Australia | Exploring family caregivers' readiness to adopt smart home technology for monitoring | 10 family caregivers (mean age = 53.3 years, 90% women) | Health-focused smart home technologies (e.g., ADL sensors, safety alerts) | Privacy, cost, fear of misuse | Awareness of benefits, reduced caregiver burden |
| Naudé et al., 2023 | France | Identifying barriers and enablers for the use of digital interactive television (DiTV) in nursing homes | 18 older adults aged 68-90 years (61% women); 6 care professionals | Smart tv system (e-lioTV): video calls, messaging, news, digital games, and home automation | Usability, privacy | / |
| Braspenning et al., 2022 | Netherlands | Implementation of lifestyle monitoring with infrared sensors to support older adults living alone | 14 participants (11 women; caregivers, healthcare professionals, and managers) | Passive infrared sensors for movement and alerts | Poor functionality, usability | Tailored training |
| Glomsås et al., 2022 | Norway | Ethical aspects of welfare technology in home care | 18 family caregivers (8 men, 10 women, aged 54-77; spouses, children etc) | Safety alarms, GPS devices, digital dispensers, digital locks and calendars | Lack of human interaction, lack of knowledge, usability | / |
| Kavčič et al., 2023 | Slovenia | Post-implementation acceptance of home-based e-care services | 7 dyads (caregivers: older adults; mean age 80) | HBECS: motion sensors, emergency buttons, fall detectors, mobile apps | Privacy, poor functionality cost | Tailored training, user-centred design |
| Jahnke et al., 2021 | USA | Adoption challenges of an alert system in assisted living | 5 nursing staff (facility manager, social workers, LPN, front-line staff) | Sensor-based alert system (motion, depth, bed sensors) | Poor functionality, lack of knowledge | Awareness of benefits, tailored training |
| Read et al., 2022 | Canada | Stakeholder perspectives on in-home passive monitoring | 28 interviews with older adults, caregivers, and social workers | Motion sensors, cameras, medication adherence monitors | Privacy, perceived lack of need | Awareness of benefits, |
| Dequanter et al., 2022 | Belgium | Technology adoption and continued use by cognitively impaired older adults | 16 older adults with mild cognitive impairment (mean age: 76) and 8 caregivers | Monitoring devices, | Usability, lack of human interaction | Caregiver buy-in |
| Bin Noon et al., 2023 | Canada | Role of active assisted living (AAL) technologies in aging in place | 18 stakeholders (care recipients, caregivers, tech developers) | AAL systems (e.g., IoT health monitors, motion sensors) | Privacy, cost | awareness of benefits |
| Taylor et al., 2022 | UK | User-centered design of digital telecare for independent living in the community | 74 participants (13 end-users: 77% female, mean age ~75; 32 informal carers; 29 H&SC professionals) | Smart home systems (e.g., alarms, GPS trackers, fall detection systems) | Stigma, usability, fear of improper use | User-centred design |
| Halvorsrud et al., 2023 | Norway | Perspectives on assistive technology among home-dwelling older adults | 7 interviewees (aged 65+, receiving community care) | Social alarms, tablet PCs, mobility aids | Lack of human interaction, usability, privacy | Tailored training, user-centred design, awareness of benefits |
| Nallam et al., 2020 | USA | Benefits of intelligent voice assistants (IVAs) | 10 low-income older adults (60-76 years, mean age 67). | Intelligent Voice Assistants (IVAs) | Privacy, usability | Awareness of benefits |
| Peek et al., 2016 | Netherlands | Comprehensive model for aging in place | 53 participants (aged 68-95, mean age 78; 64% women; varied health statuses) | ICT devices, personal alarms, mobility aids | Privacy, usability, cost, lack of human interaction | Tailored training, awareness of benefits |
| Blocker et al., 2023 | USA | Training needs and usability of voice-activated digital home assistants (DHAs) | 7 older adults (mean age 76; 60% female; 4 with disabilities; no prior DHA experience) | Smart speaker | Poor functionality, usability | Tailored training, user-centred design |
| Gordon et al., 2022 | Australia | Evaluating the effectiveness of a co-designed technology package on safety perceptions and well-being | 30 participants (mean age = 79 years; 73% female, 83% living alone; 50% had a low fall risk) | Fall detection monitors, digital assistants, memory compensatory devices | Cost, lack of knowledge, usability, stigma | Tailored training, user-centred design |

**Supplementary Table S3: Studies of Telehealth, mHealth, and Communication Tools**

| **Author & Year** | **Country** | **Main Focus** | **Participants** | **Technologies** | **Barriers** | **Facilitators** |
| --- | --- | --- | --- | --- | --- | --- |
| Blok et al., 2020 | Netherlands | Barriers and benefits of ICT for older adults | 35 older adults with cognitive impairments and network members (majority women) | ICTs for relationships, hobbies, daily activities | Usability, | Awareness of benefits |
| Cimperman et al., 2013 | Slovenia | Perceptions of home telehealth services | 12 focus groups with older adults (urban and rural areas) | Home telehealth services (health record access, assisted living, remote monitoring) | Fear, privacy, lack of knowledge | Awareness of benefits |
| Nahm et al., 2020 | USA | Older adults’ experiences with patient portals | 138 older adults (50-92 years, 67.4% women, chronic conditions). | Patient portals | Usability, privacy, lack of knowledge | Tailored training |
| Newbould et al., 2021 | England | Videoconferencing for healthcare in care homes | 25 participants (residents aged 65+ and care staff across 3 care homes). | Videoconferencing platforms | Lack of knowledge. | Tailored training |
| Shah et al., 2013 | USA | Telemedicine-enhanced emergency care programs | 21 participants (median age 92; patients, caregivers, CTAs, telemedicine providers) | Telemedicine platforms (digital otoscopes, high-res cameras) | Lack of knowledge, poor functionality | Awareness of benefits |
| Pirhonen et al., 2020 | Finland, Ireland | Perceptions of digital tech’s impact on well-being | 86 older adults (aged 55-101) | Digital platforms (telecommunication, e-health tools, internet banking) | Lack of knowledge, usability | Reduced caregiver burden |
| Mishuris et al., 2014 | USA | Barriers and facilitators of a patient portal | 14 veterans (aged 50-100, all men, mostly non-Hispanic White) receiving home care | Electronic patient portal | Usability, perceived lack of need | / |
| Vergouw et al., 2020 | Netherlands | eHealth applications for chronic condition management | 19 participants (aged 60-85, mean 73, 47% women) | eHealth apps for appointments, prescriptions, health records | Usability, lack of human interaction | Reduced caregiver burden, awareness of benefits |
| Oche et al., 2022 | USA | Factors impeding telemedicine for older adults | 33 participants (3 patients aged 65-85, caregivers, physicians, admin staff) | Telemedicine video platforms | Poor functionality, usability | Tailored training |
| Qi Tan et al., 2022 | Singapore | Barriers to nurse-facilitated teleconsultations | 22 nursing home nurses (86.3% women, varied long-term care experience) | Telemedicine platforms | Poor functionality, usability | Tailored training, awareness of benefits |
| Pogorzelska-Maziarz et al., 2021 | USA | Usability and acceptability of on-demand telehealth services | 30 participants (15 patients, 10 healthcare staff, 5 caregivers) | telehealth services | privacy, cost, usability | Awareness of benefits, support |
| Conway et al., 2023 | UK | Exploring factors influencing mobile app adoption for people with dementia | 15 individuals aged 60-90 years (7 women, 8 men); varying experience with tech | Mobile applications for communication and daily activities | Usability, fear of misuse, privacy, lack of human interaction | Support, awareness of benefits |
| Young et al., 2023 | USA | Design and evaluation of a cognitive screening app | 11 stakeholders, five older adults (aged 65-78, 60% women). | MyCog Mobile: smartphone app for remote cognitive screening | Usability, lack of knowledge | User-centred design, support |
| Ladekjær Larsen et al., 2022 | Denmark | Motion-based training (MBT) technology for home rehabilitation in persons with dementia | Four individuals with dementia (aged 67-82, 75% men) and four relatives, three healthcare professionals | MBT device with touch screen, Kinect camera, modem | usability, lack of human interaction, perceived lack of need | Reduced carer burden |
| Chan-Nguyen et al., 2022 | Canada | Barriers and solutions for virtual care | 18 participants (aged 29-94; 67% women, 39% disabled) | Virtual care tools: video conferencing, telephone | usability, privacy, lack of human interaction | support |
| Judson et al., 2023 | USA | Older adults’ perceptions of e-visits | 20 older adults (>65 years, diverse backgrounds, varying tech literacy) | Patient portal e-visits | Lack of human interaction, lack of knowledge, | / |
| Gately et al., 2022 | USA | In-home video telehealth for dementia management | 24 caregivers of older veterans with dementia | In-home video telehealth platforms | Lack of knowledge, usability, | Tailored training |
| Dukhanin et al., 2023 | USA | Increasing shared access to patient portals | Stakeholders across 3 healthcare organisations, including older adults, caregivers, and healthcare providers | Patient portals | Poor functionality, lack of knowledge | User-centred design, tailored training |
| Mao et al., 2022 | USA | Barriers to telemedicine for older adults in independent living facilities | 15 older adults | Computers, smartphones, tablets | Lack of knowledge, usability | Support, tailored training |
| Xiang et al., 2021 | USA | Internet-based psychotherapy for depression in homebound older adults | 21 homebound older adults (81% women, mean age 76) | Internet-based consultations | Usability, lack of knowledge, | Support, user-centred design |
| Cha et al., 2022 | USA | Personalised recommendations for dementia caregivers via a web-based tool | 20 dementia caregivers guided by senior care navigators | web-based treatment planning tool | Cost, lack of knowledge, usability | support |
| Piculell et al., 2021 | Sweden | Experiences of older adults with cognitive impairment using mobile health apps | 16 participants (aged 71-100, 25% women) with mild cognitive impairment | SMART4MD app (medication reminders, cognitive games) | Usability | User-centred design, support |
| Hawley-Hague et al., 2021 | UK | Feasibility and usability of teleconferencing for falls prevention exercises | 7 older adults (60+ years), 3 health professionals | Teleconferencing software | Poor functionality, cost, perceived lack of need | Support |
| Arkkukangas et al., 2021 | Sweden | mHealth for fall prevention: usability and behaviour change strategies | 12 older adults (70-83 years, 58% female), familiar with various technological devices | mobile app for fall prevention (strength and balance exercises) | Usability, poor functionality | / |
| Hunter et al., 2022 | New Zealand | Barriers and enablers of telehealth for rural older adults | 98 participants (69% women, aged 55-92; 30% Māori) | Telehealth platforms (phone, video consultations, patient portals) | Poor functionality, cost, lack of knowledge | User-centred design |
| Albers et al., 2022 | USA | Technology use among persons with memory concerns and caregivers | 20 dyads (PWMCs: mean age 74.8 years, 70% men and their carers | teleconferencing, smartphones, fitness trackers, medical alarms | Lack of human interaction, lack of knowledge | Reduced caregiver burden |
| Alexander et al., 2021 | USA | VVC telehealth program for enhancing post-discharge mobility in older veterans | 45 veterans (mean age 73 years) with mobility and cognitive impairments | VA Video Connect (VVC) | Poor functionality, usability, lack of human interaction | Support |
| Amiri et al., 2023 | Iran | Electronic health record adoption factors for Alzheimer’s care | 13 caregivers and 7 neurologists | Electronic personal health records | Privacy, lack of knowledge, usability | User-centred design |
| Hawley et al., 2024 | USA | Barriers and facilitators of video visits for older adults | 20 participants aged 68-80 years (Mean = 74, 95% men), predominantly non-Hispanic White, with chronic conditions | VA-loaned tablets, desktops, laptops connected to VA’s video platform | Poor functionality, | Support |
| Lee et al., 2023 | UK | Barriers and facilitators to online/app use for dementia support | 47 participants with dementia (aged 65-84, 72.3% men) and 62 carers | Computers, laptops, smartphones, communication apps | Lack of knowledge, privacy, usability | Awareness of benefits |
| Nymberg et al., 2019 | Sweden | Beliefs and expectations of e-health in primary healthcare | 15 elderly patients (aged 65-80, 8 women) with chronic conditions | E-health services (online booking, video consultations, health monitoring tools) | privacy, lack of knowledge, cost, poor functionality | Support, awareness of benefits |
| Patel et al., 2022 | Australia | Implementation of a digital tool to enhance social engagement | 33 participants (17 staff and 30 students, including allied health students) | social network tool (digital and paper-based platform) | Lack of knowledge, usability, privacy | Tailored training |
| Smith et al., 2022 | USA | Acceptability of patient portal for improving communication in primary care | 22 patient-family dyads (average age = 82.3 years, 72.7% women), 30 stakeholders (clinicians, caregivers) | Shared patient portal access | Usability, lack of knowledge, perceived lack of need | Support |
| Vaportzis et al., 2017 | UK | Older adults' perceptions and attitudes toward tablet computers | 18 older adults (aged 65-76 years, 83.3% women) | Tablets | Lack of knowledge, usability, cost | Tailored training, awareness of benefit, support |
| Fothergill et al., 2023 | UK | Value of proactive telecare systems for independence at home | 30 participants: 15 telecare users (mean age: 74.6 years), 5 non-users (mean age: 74.2 years), family members, staff | proactive telecare system (tablet-based check-ins linked to a call centre) | Cost, perceived lack of need, lack of human interaction | Awareness of benefits |
| Hoel et al., 2022 | Germany | Feasibility and usability of the I-CARE system | 9 dyads (PLWD: mean age 77, caregivers: mean age 72; mostly men PLWD and women caregivers) | Tablet-based activation system (I-CARE) | usability, perceived lack of need | Tailored training, awareness of benefits |
| Tsertsidis, 2021 | Sweden | Challenges in delivering digital technologies for aging in place (DTAP) to individuals with dementia | Key stakeholders (4 occupational therapists, 3 caseworkers, 1 researcher, 3 relatives) | Digital technologies for aging in place (e.g., electronic calendars, mobile devices, apps) | usability, lack of knowledge, cost | User-centred design |
| Castro Rojas, 2021 | Costa Rica | Barriers and supports for older adults learning ICT | 41 older adults (mean age 67.15, 80% women) | ICT devices and platforms (laptops, smartphones, cognitive training games) | Lack of knowledge, usability | Support, tailored training |
| Essery et al., 2021 | UK | Digital intervention for cognitive health | 59 older adults (mean age ~70, mix of high and low cognitive performance) | Active Brains (cognitive training, physical activity, healthy eating) | Usability, lack of knowledge, perceived lack of need | Support, user-centred design |
| Böttinger et al., 2023 | Germany | Co-creation of a digital self-assessment system for older adults | 10 older adults (mean age 77.8 years, 60% women), all with prior ICT experience | Tablet-based self-assessment apps | Lack of knowledge, fear of misuse | Awareness of benefits |
| Thach et al., 2023 | Australia | Technology design for social enrichment in residential aged care facilities | Residents and staff at Vietnamese-Australian aged care home | Video-calling systems, virtual reality (VR) | Poor functionality, usability | User-centred design |
| Halvorsrud et al., 2023 | Norway | Perspectives on assistive technology among home-dwelling older adults | 7 interviewees (aged 65+, receiving community care) | Social alarms, tablet PCs, mobility aids | Lack of human interaction, usability, privacy | Tailored training, user-centred design, awareness of benefits |
| Peek et al., 2016 | Netherlands | Comprehensive model for aging in place | 53 participants (aged 68-95, mean age 78; 64% women; varied health statuses) | ICT devices, personal alarms, mobility aids | privacy, usability, cost, lack of human interaction | Tailored training, awareness of benefits |
| Garcia Reyes et al., 2023 | Australia | Older adults' experiences with health self-management technologies | 22 older adults (64% female, aged 65-87, mean age 73) | Telehealth, fitness trackers, medical devices | usability, privacy, cost | User-centred design |

**Supplementary table S4: Studies of Workforce Support Tools**

| **Author & Year** | **Country** | **Main Focus** | **Participants** | **Technologies** | **Barriers** | **Facilitators** |
| --- | --- | --- | --- | --- | --- | --- |
| Curtis & Brooks, 2020 | England | Implementation of digital health tech in nursing homes | 20 participants (nursing home staff, residents, relatives) | Digital health tools (telehealth, wearables, digital records) | Privacy, perceived lack of need, usability, | Tailored training, user-centred design |
| Faisal et al., 2020 | Canada | Stakeholder feedback on medication adherence devices | 37 participants (57% older adults, 14% caregivers, 30% healthcare providers) | Medication adherence devices (alarms, portability, cloud connectivity) | usability, cost | / |
| Munck et al., 2012 | Sweden | Medical tech in palliative home care | 15 next-of-kin caregivers (39-83 years, 60% women). | Medical telecare devices | Poor functionality, perceived lack of need | Awareness of benefits |
| Hafdi et al., 2021 | UK, China | Development of PRODEMOS mHealth platform | 77 participants (aged 55+; UK: 21, China: 56), interviews and focus groups with older adults and developers | mHealth platform for dementia prevention | Lack of knowledge, poor functionality, usability | Tailored training |
| Di Pelino et al., 2022 | Canada | Development and implementation of the a digital staff support tool | Stakeholders: primary care providers, volunteers, volunteer coordinators, older adults | web-based app for volunteer-client management, surveys, and care plans | Poor functionality, lack of knowledge, usability | User-centred design |
| Kupka-Klepsch et al., 2024 | Austria | Evaluation of digital documentation and training system | 25 caregivers, nurses, and relatives | e-documentation, e-learning, emergency management) | Lack of knowledge | User-centred design |
| Qi et al., 2023 | China | Barriers to internet-based home care services for older adults from the provider perspective | 34 staff members from 14 Home Care Service Centres (70.6% women, mean 3.9 years’ experience) | Internet-based Home Care Services | usability, cost, privacy, lack of knowledge | / |
| Kabacińska et al., 2023 | Canada | Older adults' priorities for the evaluation of assistive technologies | 21 AT users (62% women, aged 50+, experience with mobility devices, hearing aids) | Various assistive technologies | Cost, stigma, usability, | / |

**Supplementary Table S5: Studies of Robots**

| **Author & Year** | **Country** | **Main Focus** | **Participants** | **Technologies** | **Barriers** | **Facilitators** |
| --- | --- | --- | --- | --- | --- | --- |
| Zsiga et al., 2013 | Austria, France, Hungary | User attitudes toward socially active robots | 3 focus groups (older adults with impairments, caregivers) | Domeo social robots (AAL Joint Programme) | Privacy, lack of human interaction | Awareness of benefits |
| Dosso et al., 2023 | Canada | Emotional alignment and adoption of social robots | 44 participants: 25 older adults (aged 50-80+; 20 women), 17 care partners | Pet-like social robots | Stigma, privacy, poor functionality | Awareness of benefits |
| Johansson-Pajala & Gustafsson, 2022 | Sweden | Challenges of introducing care robots in elder care | 21 participants (advisors, project leaders, representatives of robotics companies and public organizations; 67% women, aged 37-79) | Care robots for assistance, monitoring, and companionship | privacy, costs, usability, lack of knowledge | Tailored training, user-centred design |
| Zafrani et al., 2024 | Israel | Older adults' perceptions of socially assistive robots | 19 older adults (mean age= 81 years, 53% women; mostly community-dwelling and retired) | Humanoid socially assistive robot for physical and cognitive training | Lack of knowledge, privacy, lack of human interaction | Tailored training |
| Kang et al., 2023 | South Korea | Nurses’ perceptions of care robots and experiences | 18 nurses (mean age = 42.8 years, 94% women) | AI-based socially assistive robots | Poor functionality, usability, | Awareness of benefits |
| Sawik et al., 2023 | Poland, Spain | Use of social and service robots in elderly care | Older adults with mild cognitive impairment (MCI), caregivers | Socially assistive robots (SARs) | Usability, privacy, cost | Support, awareness of benefits |
| Wong et al., 2023 | Canada | Implementation of telepresence robots in long-term care (LTC) | 22 care staff (nurses, care aides, allied health practitioners) | Telepresence robots for virtual family visits | Lack of knowledge, poor functionality | Tailored training, awareness of benefits |
| Arthanat et al., 2024 | USA | Perspectives on telepresence robots in independent living facilities | 10 stakeholders: 3 residents (aged 65+), 3 ambassadors, 2 staff, 2 occupational therapy students | telepresence robot | Poor functionality, usability, cost, lack of human interaction | support |
| Mahmoudi Asl et al., 2023 | Netherlands, Spain | Implementation of MINI robot in meeting centres for people with dementia | 12 older adults with mild-to-moderate dementia, 11 stakeholders (managers, therapists, policymakers) | Interactive humanoid robot | Lack of human interaction, perceived lack of need | tailored training |
| Waycott et al., 2022 | Australia | Technology-mediated enrichment in aged care | 12 care staff (care assistants, technology providers) | VR, robot pets, videoconferencing, tablets | cost, perceived lack of need, lack of knowledge | / |

**Supplementary Table S6: Supporting quotes for qualitative findings**

| **Theme name** | **Study ID** | **Quote** | **Technology type** | **Participant descriptor** |
| --- | --- | --- | --- | --- |
| Usability | Conway 2023 | As long as what’s on the app [can be kept] simple. It’s keeping it simple and keeping it workable for me  but it’s not that easy being workable for me. I feel sometimes it can be overwhelming. | Tele/mHealth | Older adult, male |
|  | Glomsas 2022 | The medication dispenser was initially put in my mother’s living room by the healthcare professionals. But she needs to reach the medication while she is still in bed | Smart home technologies | Relative of older adult |
|  | Kang 2023 | They (older adults) keep forgetting to recharge it. When they said it (care robot) was not working or talking, most of the time, it was because they did not recharge it, or it was turned off, or the volume was very low, or they were trying to plug the charger into the wrong port, or not using the specific robot cable that fits. | Robot | Care provider |
| Poor functionality | Hunter 2022 | ...it’s intermittent...I think I’ve sent that email and no,...I haven’t or, I thought I had paid that bill, and no I hadn’t paid it [because the connection dropped  off] | Tele/mHealth | Older adult |
|  | Arthanat 2023 | Each time I took [robot] to an  apartment there would be a disconnect and I would have to reconnect | Robot | Care staff |
|  | Kang 2023 | …replacing it with another when the device  breaks down, teaching and answering questions, reconnecting it again when Bluetooth is not working,  and so on. They quit because there is too much work involved [in maintaining the technology] | Robot | Care staff |
|  | Chaudhuri 2015 | And then it went off once [wearable alarm], and uh, and one of the other ladies that had used it before finally got it to go off, but then she handed it back to me and I just laid it down again and it went off again. | Wearables | Older adult |
| Discomfort | Chaudhuri 2015 | I found it heavy to wear around my neck, um,  little uncomfortable, and um, wondering you know  whether there was something lighter that would be used instead of that would still accomplish what you wanted to. | Wearables | Older adult |
|  |  |  |  |  |
| Cost | Qi 2023 | There are a lot of older adults who need care services. It seems there is much demand. However, they do not meet the eligibility criteria for a free Home Care Service Voucher, and the elderly have low pensions, so few of them are willing to buy it | Workforce support tools | Care centre manager |
|  | Neubauer 2022 | The income is not as fluid as it was, and so you’re very fixed. So cost is a big deal | Wearables | Carer of older adult |
|  | Elers 2018 | A lot of elderly people, they’ve got very little  money…who’s going to pay for it? | Smart home technology | Family member of older adult |
|  | Fothergill 2023 | Well, it’s not free, is it, that’s the thing. And until you  need it [proactive telecare], I guess you don’t realise  it’s important...and I think a lot of people probably  put it off | Tele/mHealth | Family member of older adult |
| Lack of Knowledge | Glomsas 2022 | She has a safety alarm but does not know how to use it. She does not understand… she no longer thinks about the fact that she might need help | Smart home technology | Relative of older adult |
|  | Mahmoudi Asl 2023 | I cannot leave those people alone. Because they don’t know what to do [with the robot]. You always have to be with them. | Robot | Therapist |
|  | Orellano-Colon 2016 | knowing how to use them [smart home devices], because otherwise, it would not have a positive effect. | Smart home technology | Older adult |
|  | Pirhonen 2020 | I believe that I would need to take a course to understand thoroughly what this is all about. I utilized these technologies when I was at work, but then there always are new concepts and words, for  example an account. I’m used to understanding that accounts are for money, but in computer language, account means something else. I get annoyed when I don’t know | Tele/mHealth | Older adult |
| Fear of misuse | Dermody 2023 | I would like the requirements for other family members to have it [access to the data collected] carefully monitored because, not in our situation it's the  reverse, but I know that in some family situations, the family is quite pushing for people to go into care [nursing home]. So, I wouldn't like to think that if they had access to the print data that you're receiving that they might use that data to force someone into care | Smart home technologies | Spouse of cared -for older adult |
| Perceived lack of need | Conway 2023 | You have to have the need for the app first otherwise what’s the point? | Tele/mHealth | Older adult, male |
|  | Mahmoudi Asl 2023 | Playing games! People can do that anyway; they don’t need the robot for that. | Robot | Older adult with dementia |
|  | Davenport 2012 | I don’t need anything except for the remote control that I have for my TV. There isn’t anything else that I would need to have done [to my home]. There is nothing there, I don’t need a voice control thermostat or lights or blinds... . I don’t see, in my case anyway, any value in that [voice recognition]... .  I can’t foresee that at all in my future unless I became a quadriplegic, that’s who this would benefit, maybe a paraplegic | Smart home technology | Older adult (female) |
| Lack of human interaction | Chan-Nguyen 2022 | I don’t know, it’s just an assumption ‘cause you don’t see the person and then another thing, you don’t know if the person [care provider] is talking to you attentively. They might be doing something on computer and then talking to you on the side, so there’s always this hesitant … I don’t know | Tele/mHealth | Older adult (patient) |
|  | Arthanat 2023 | Maxine [robot] worked for the intended purposes, but I would still prefer in person | Robot | Older adult |
|  | Judson 2023 | You’re relying upon your narrative. You have to express in [the patient portal] within a certain amount of words exactly what the issue is. And believe me, that sometimes can't be captured [online]. | Tele/mHealth | Older adult (patient) |
|  | Mahmoudi Asl 2023 | I am afraid that you will lose contact with real people a bit if you constantly fixate on the robot…it helps you, it looks nice, and then what? Will you then get on better with other people? | Robot | Older adult with dementia |
| Privacy | Dermody 2023 | The only other barrier I could see is cost. I mean, I don't know how much it costs, but I can't imagine that it's a cheap buy. And then there's ongoing costs. | Smart home Technologies | Family caregiver |
|  | Chan-Nguyen 2022 | I think the issue of privacy comes up to my mind…, if you’re at home and you have other family at home and maybe you want to have a private conversation,  your family might listen in and you don’t want them to. That impacts things | Tele/mHealth | Older adult (patient) |
|  | Qi 2023 | We are involved as a third-party organization. People don’t recognize us, thinking we are liars. When we ask to use older adults’ mobile phones to register and fill in their basic information, they refuse to comply | Workforce support tools | Care centre manager |
|  | Neubauer 2022 | You’re going to get a lot of flak from people living with dementia because we are already feeling very  threatened…and will feel like [we’re] being spied on | Wearables | Older adult with dementia |
| Stigmatisation | Chaudhuri 2015 | I was in a meeting and it went off [wearable alarm] and that was kind of embarrassing | Wearables | Older adult |
|  | Neubauer 2022 | [My mom] doesn’t want to show people that she’s sick… it should be as hidden as possible… Many  [people with dementia] are very stubborn. The less sometimes they know the better | Wearables | Family member of older adult |
|  | Orellano-Colon 2016 | People could make fun of me [for having smart home technology] | Smart home technologies | Older adult |
| Awareness of benefits | Kang 2023 | Older adults with dementia need to take dementia medications. We tell them to take medicine regularly over the phone, but it is not easy to remind them every day. When the care robot is used to tell the older adult when to take medicine every morning and evening, the older adult’s awareness of taking medicine is increased by repeatedly listening to the care robot | Robot | Nurse |
|  | Davenport 2012 | I suppose, yes, because sometimes our subjective perceptions aren’t as objective assessment of what we think we are doing, yeah, yeah, yeah I agree, this [monitoring technology] could be enlightening because sometimes what we think we are doing is not really what we are doing ... . I suppose that would be useful | Smart home technology | Older adult |
| Targeted training or support | Conway 2023 | So my family has set that up for me, so it’s important that we have that support. | Tele/mHealth | Older adult, male |
|  | Hunter 2022 | bringing the people to a central hub and actually educating them,... in a community, the buy-in comes  from the community, the capacity comes from the community... | Tele/mHealth | Older adult |

**Supplementary Table S7: Quality assessment scoring**

| **CASP Statements** | 1. Was there a clear statement of the aims of the research?  2. Is a qualitative methodology appropriate?  3. Was the research design appropriate to address the aims  of the research?  4. Was the recruitment strategy appropriate to the aims of  the research?  5. Was the data collected in a way that addressed the  research issue?  6. Has the relationship between researcher and participants  been adequately considered?  7. Have ethical issues been taken into consideration?  8. Was the data analysis sufficiently rigorous?  9. Is there a clear statement of findings?  10. How valuable is the research? | | | | | | | | | |
| --- | --- | --- | --- | --- | --- | --- | --- | --- | --- | --- |
| **Author & Year** | **1** | **2** | **3** | **4** | **5** | **6** | **7** | **8** | **9** | **10** |
| Parker 2013 | Yes | Yes | Yes | Yes | Yes | No | Can't tell | Can't tell | Yes | Yes |
| Bian 2021 | Yes | Yes | Yes | Can't tell | Yes | No | Can't tell | Can't tell | Yes | Yes |
| Block 2020 | Yes | Yes | Yes | Yes | Yes | No | Can't tell | Can't tell | Can't tell | Yes |
| Cimperman 2013 | No | Can't tell | Can't tell | Can't tell | Yes | No | Can't tell | No | No | Yes |
| Cohen 2016 | Yes | Yes | Yes | Yes | Yes | No | Can't tell | Can't tell | Yes | Yes |
| Curtis 2020 | Yes | Can't tell | Can't tell | Yes | Can't tell | No | Yes | Can't tell | Yes | Yes |
| Davenport 2012 | Yes | Yes | Yes | Yes | Yes | No | Can't tell | Yes | Yes | Yes |
| Thilo 2016 | Yes | Yes | Yes | Yes | Yes | No | Can't tell | Can't tell | Yes | Yes |
| Chaudhuri 2015 | Yes | Can't tell | Yes | Yes | Yes | No | Can't tell | No | Yes | Yes |
| Elers 2018 | Yes | Yes | Yes | Yes | Yes | Can't tell | Can't tell | Yes | Yes | Yes |
| Faisal 2020 | Yes | Yes | Yes | Yes | Yes | No | Can't tell | Can't tell | Yes | Yes |
| Gately 2022 | Yes | Yes | Yes | No | Can't tell | No | Can't tell | Can't tell | Yes | Yes |
| Hall 2019 | Yes | Yes | Yes | Can't tell | Yes | Yes | Yes | Yes | Yes | Yes |
| Kononova 2019 | Yes | Yes | Yes | Yes | Yes | Can't tell | Can't tell | Yes | Yes | Yes |
| Zsiga 2013 | Yes | Yes | Yes | No | Can't tell | No | Can't tell | Can't tell | Can't tell | Yes |
| Munck 2012 | Yes | Yes | Yes | Can't tell | Yes | No | Yes | Can't tell | Yes | Yes |
| Nahm 2020 | Yes | Yes | Yes | Yes | Yes | No | Can't tell | Yes | Yes | Yes |
| Nallam 2020 | Yes | Yes | Yes | Can't tell | Yes | No | Can't tell | Can't tell | Yes | Yes |
| Newbould 2021 | Yes | Yes | Yes | Can't tell | Yes | No | Can't tell |  | Yes | Yes |
| Nymberg 2019 | Yes | Yes | Yes | Yes | Yes | No | Can't tell | Yes | Yes | Yes |
| Orellano-Colon 2015 | Yes | Yes | Yes | Yes | Yes | No | Can't tell | Yes | Yes | Yes |
| Peek 2016 | Yes | Yes | Yes | Can't tell | Yes | No | Can't tell | Can't tell | Yes | Yes |
| Pirhonen 2020 | Yes | Yes | Yes | Yes | Yes | No | Can't tell | Can't tell | Yes | Yes |
| Pol 2016 | Yes | Yes | Yes | Can't tell | Can't tell | No | Can't tell | Can't tell | Yes | Yes |
| Shah 2013 | Yes | Yes | Yes | Yes | Yes | No | Can't tell | Can't tell | Yes | Yes |
| Mishuris 2014 | Yes | Yes | Yes | Can't tell | Yes | No | Can't tell | Can't tell | Yes | Yes |
| Vaportzis 2017 | Yes | Yes | Yes | Yes | Yes | Can't tell | Can't tell | Yes | Yes | Yes |
| Vergouw 2020 | Yes | Yes | Yes | Yes | Yes | Can't tell | Yes | Yes | Yes | Yes |
| Wang 2019 | Yes | Yes | Yes | Yes | Yes | No | Can't tell | Can't tell | Yes | Yes |
| Kim et al., 2021 | Yes | Yes | Yes | Yes | Yes | Yes | Can't tell | Yes | Yes | Yes |
| Gomez et al., 2021 | Yes | Yes | Yes | Yes | Yes | No | Yes | Yes | Yes | Yes |
| Neubauer et al., 2021 | Yes | Yes | Yes | Can't tell | Yes | No | Yes | Yes | Yes | Yes |
| Dequanter et al., 2022 | Yes | Yes | Yes | Yes | Yes | No | Yes | Yes | Yes | Yes |
| Gris et al., 2023 | Yes | Yes | Yes | Yes | Yes | No | Yes | Yes | Yes | Yes |
| Garcia Reyes et al., 2023 | Yes | Yes | Yes | Yes | Yes | No | Yes | Can't tell | Yes | Yes |
| Mishra et al., 2023 | Yes | Yes | Yes | Can't tell | Yes | No | Can't tell | Can't tell | Yes | Yes |
| Gottwald et al., 2016 | Yes | Yes | Yes | Can't tell | Can't tell | No | No | Yes | Yes | Yes |
| Dermody et al., 2023 | Yes | Yes | Yes | Yes | Yes | Yes | Yes | Yes | Yes | Yes |
| Naudé et al., 2023 | Yes | Yes | Yes | Yes | Yes | No | Can't tell | Yes | Yes | Yes |
| Braspenning et al., 2022 | Yes | Yes | Yes | Yes | Yes | No | No | Yes | Yes | Yes |
| Glomsås et al., 2022 | Yes | Yes | Yes | Can't tell | Yes | Yes | Yes | Yes | Yes | Yes |
| Kavčič et al., 2023 | Yes | Yes | Yes | Can't tell | Yes | No | Yes | Yes | Yes | Yes |
| Jahnke et al., 2021 | Yes | Yes | Yes | Yes | Yes | No | Yes | Yes | Yes | Yes |
| Read et al., 2022 | Yes | Yes | Yes | Yes | Can't tell | No | Yes | Yes | Yes | Yes |
| Bin Noon et al., 2023 | Yes | Yes | Yes | Yes | Yes | No | Yes | Yes | Yes | Yes |
| Taylor et al., 2022 | Yes | Yes | Yes | Can't tell | Can't tell | No | Yes | Yes | Yes | Yes |
| Halvorsrud et al., 2023 | Yes | Yes | Yes | Can't tell | Yes | No | Yes | Yes | Yes | Yes |
| Blocker et al., 2023 | Yes | Yes | Yes | Can't tell | Can't tell | No | No | Can't tell | Yes | Yes |
| Gordon et al., 2022 | Yes | Yes | Yes | Can't tell | Yes | No | Can't tell | Can't tell | Yes | Yes |
| Oche et al., 2022 | Yes | Yes | Yes | Yes | Yes | No | No | Can't tell | Yes | Yes |
| Qi Tan et al., 2022 | Yes | Yes | Yes | Yes | Yes | No | Can't tell | Yes | Yes | Yes |
| Pogorzelska-Maziarz et al., 2021 | Yes | Yes | Yes | Yes | Yes | No | No | Yes | Yes | Yes |
| Conway et al., 2023 | Yes | Yes | Yes | Yes | Yes | Yes | Yes | Yes | Yes | Yes |
| Young et al., 2023 | Yes | Yes | Yes | Yes | Yes | Can't tell | Yes | Yes | Yes | Yes |
| Ladekjær Larsen et al., 2022 | Yes | Yes | Yes | Yes | Yes | No | Yes | Can't tell | Yes | Yes |
| Chan-Nguyen et al., 2022 | Yes | Yes | Yes | Yes | Yes | Yes | Yes | Yes | Yes | Yes |
| Judson et al., 2023 | Yes | Yes | Yes | Yes | Yes | Yes | Can't tell | Yes | Yes | Yes |
| Dukhanin et al., 2023 | Yes | Yes | Yes | Yes | Can't tell | No | Can't tell | Can't tell | Yes | Yes |
| Mao et al., 2022 | Yes | Yes | Yes | Yes | Yes | No | Can't tell | Yes | Yes | Yes |
| Xiang et al., 2021 | Yes | Yes | Yes | Yes | Yes | No | No | Yes | Yes | Yes |
| Cha et al., 2022 | Yes | Yes | Yes | Yes | Yes | No | Can't tell | Yes | Yes | Yes |
| Piculell et al., 2021 | Yes | Yes | Yes | Yes | Yes | No | Yes | Yes | Yes | Yes |
| Hawley-Hague et al., 2021 | Yes | Yes | Yes | Yes | Yes | No | Yes | Yes | Yes | Yes |
| Arkkukangas et al., 2021 | Yes | Yes | Yes | Yes | Yes | No | Can't tell | Yes | Yes | Yes |
| Hunter et al., 2022 | Yes | Yes | Yes | Yes | Yes | Yes | Yes | Yes | Yes | Yes |
| Albers et al., 2023 | Yes | Yes | Yes | Yes | Yes | Can't tell | Can't tell | Yes | Yes | Yes |
| Alexander et al., 2021 | Yes | Can't tell | Yes | Yes | Can't tell | No | No | No | Yes | Yes |
| Amiri et al., 2023 | Yes | Yes | Yes | Yes | Yes | No | No | Yes | Yes | Yes |
| Hawley et al., 2024 | Yes | Yes | Yes | Yes | Yes | No | No | Yes | Yes | Yes |
| Lee et al., 2023 | Yes | Yes | Yes | Yes | Can't tell | No | Yes | No | Yes | Yes |
| Patel et al., 2022 | Yes | Yes | Yes | Yes | Yes | Can't tell | Yes | Yes | Yes | Yes |
| Smith et al., 2022 | Yes | Yes | Yes | Yes | Yes | No | No | Yes | Yes | Yes |
| Fothergill et al., 2023 | Yes | Yes | Yes | Yes | Yes | No | Yes | Yes | Yes | Yes |
| Hoel et al., 2022 | Yes | Yes | Yes | Yes | Yes | No | Yes | Yes | Yes | Yes |
| Tsertsidis, 2021 | Yes | Yes | Yes | Yes | Yes | No | No | Yes | Yes | Yes |
| Castro Rojas, 2021 | Yes | Yes | Yes | Yes | Can't tell | No | No | Can't tell | Yes | Yes |
| Essery et al., 2021 | Yes | Yes | Yes | Yes | Yes | No | Can't tell | Yes | Yes | Yes |
| Böttinger et al., 2023 | Yes | Yes | Yes | Yes | Yes | No | Yes | Yes | Yes | Yes |
| Thach et al., 2023 | Yes | Yes | Yes | Yes | Yes | No | Can't tell | Can't tell | Yes | Yes |
| Hafdi et al., 2021 | Yes | Yes | Yes | Yes | Yes | No | Yes | Yes | Yes | Yes |
| Di Pelino et al., 2022 | Yes | Can't tell | Can't tell | Yes | Can't tell | No | No | Can't tell | Yes | Yes |
| Kupka-Klepsch et al., 2024 | Yes | Yes | Yes | Yes | Yes | No | Yes | Yes | Yes | Yes |
| Qi et al., 2023 | Yes | Yes | Yes | Yes | Yes | Yes | Yes | Yes | Yes | Yes |
| Kabacińska et al., 2023 | Yes | Yes | Yes | Yes | Yes | No | Yes | Yes | Yes | Yes |
| Dosso et al., 2023 | Yes | Yes | Yes | Yes | Yes | Can't tell | Yes | Yes | Yes | Yes |
| Johansson-Pajala & Gustafsson, 2022 | Yes | Yes | Yes | Yes | Yes | No | Yes | Yes | Yes | Yes |
| Zafrani et al., 2024 | Yes | Yes | Yes | Yes | Yes | No | Yes | Yes | Yes | Yes |
| Kang et al., 2023 | Yes | Yes | Yes | Yes | Yes | No | Can't tell | Yes | Yes | Yes |
| Sawik et al., 2023 | Yes | Yes | Yes | Yes | Yes | No | Yes | Yes | Yes | Yes |
| Wong et al., 2023 | Yes | Yes | Yes | Yes | Yes | No | Yes | Yes | Yes | Yes |
| Arthanat et al., 2024 | Yes | Yes | Yes | Yes | Yes | Yes | Can't tell | Yes | Yes | Yes |
| Mahmoudi Asl et al., 2023 | Yes | Yes | Yes | Yes | Yes | No | Yes | Can't tell | Yes | Yes |
| Waycott et al., 2022 | Yes | Yes | Yes | Yes | Yes | Can't tell | Yes | Yes | Yes | Yes |

**Supplementary table S8: Excluded full text articles with reasons**

| Study title | Study ID | Reason for Exclusion |
| --- | --- | --- |
| "I wouldn't have joined if it wasn't online": understanding older people's engagement with teleyoga classes for fall prevention | Haynes 2022 | technology does not support care |
| A Multi-Site Trial of the Impact of Assistive Technology With Assistance Users and Their Caregivers | NCT01640470 2011 | barriers/facilitators not explored in a meaningful way |
| A study to test the effectiveness of an internet-based program to support caregivers of persons with dementia | CTRI/2017/02/007876 2017 | barriers/facilitators not explored in a meaningful way |
| An In-Home Advanced Robotic System to Manage Elderly Home-Care Patients' Medications: a Pilot Safety and Usability Study | Rantanen 2017 | barriers/facilitators not explored in a meaningful way |
| Client Satisfaction with Telehealth in Assisted Living and Homecare | Grant 2015 | barriers/facilitators not explored in a meaningful way |
| Closing the Digital Divide in Speech, Language, and Cognitive Therapy: cohort Study of the Factors Associated With Technology Usage for Rehabilitation | Munsell 2020 | barriers/facilitators not explored in a meaningful way |
| Effect of robot for medication management on home care professionals' use of working time in older people's home care: a non-randomized controlled clinical trial | Kajander-Unkuri 2023 | barriers/facilitators not explored in a meaningful way |
| Effectiveness of a Smart Hearing Aid on Improving Psychosocial Well-being in Elderly | NCT05772949 2023 | Abstract |
| Effectiveness of Home Modification on Falling, Occupational Performance and Life Habits in Elderly with Dementia | IRCT20210106049950N1 2021 | Wrong study design |
| Empowering Elders Through Technology | NCT00261664 2005 | barriers/facilitators not explored in a meaningful way |
| Engagement and clinical improvement among older adult primary care patients using a mobile intervention for depression and anxiety: case studies | CaseyOrr 2020 | Wrong study design |
| Evaluating the effectiveness of a social robot intervention on pain management in people with dementia living in residential aged care facilities: a cluster randomised controlled trial | ACTRN12621001010886 2021 | Abstract |
| Feasibility and Preliminary Efficacy of Two Technology-assisted Vocal Interventions for Older Adults Living in a Residential Facility | Johnson 2022 | Wrong study design |
| Feasibility, usability, and acceptance of â€œBrain-ITâ€â€”A newly developed exergame-based training concept for the secondary prevention of mild neurocognitive disorder: a pilot randomized controlled trial | Manser 2023 | barriers/facilitators not explored in a meaningful way |
| Impacts of a care robotics project on finnish home care workersâ€™ attitudes towards robots | Rantanen 2020 | barriers/facilitators not explored in a meaningful way |
| Incorporating technology in research with older bereaved adults: lessons learned from conducting an internet-based randomized controlled trial | Lyew 2023 | barriers/facilitators not explored in a meaningful way |
| MOTIVATE: moving to Improve Outcomes for Older Adults | NCT03914469 2019 | Wrong study design |
| Older patients' use of technology for a post-discharge nutritional intervention - A mixed-methods feasibility study | Lindhardt 2017 | barriers/facilitators not explored in a meaningful way |
| Process Evaluation of a Vanguard Phase of a Trial for a Smartphone- App Based Model Of Care | Redwood 2022 | Abstract |
| Tailoring digital apps to support active ageing in a low income community | Gomes 2019 | Wrong setting |
| The acceptability, adoption, and feasibility of a music application developed using participatory design for home-dwelling persons with dementia and their caregivers. The â€œAlightâ€ app in the LIVE@Home.Path trial | Berge 2022 | barriers/facilitators not explored in a meaningful way |
| The Brain Health Champion study: a health coaching intervention with mobile technology in older adults with mild cognitive impairment or risk factors for dementia | Krivanek 2021 | Abstract |
| Usefulness of navigation application for outdoor mobility guides in community-dwelling older adults: a preliminary study | Shimokihara 2021 | barriers/facilitators not explored in a meaningful way |
| "Are we ready for robots that care for us?" Attitudes and opinions of older adults toward socially assistive robots | Pino 2015 | barriers/facilitators not explored in a meaningful way |
| "Do i need it? Do i really need it?" Elderly peoples experiences of unmet assistive technology device needs | Storli 2013 | barriers/facilitators not explored in a meaningful way |
| "Grandma, You Should Do It-It's Cool" Older Adults and the Role of Family Members in Their Acceptance of Technology | Luijkx 2015 | barriers/facilitators not explored in a meaningful way |
| "How do you care for technology?" - Care professionals' experiences with assistive technology in care of the elderly | Saborowski 2015 | barriers/facilitators not explored in a meaningful way |
| "I Want to Keep the Personal Relationship With My Doctor": Understanding Barriers to Portal Use among African Americans and Latinos | Lyles 2016 | Wrong patient population |
| "if My Doctors Had Recommended a Mobile App to Me Back Then, i Wouldn't Have Been in This Situation Today": Identifying the Barriers and Facilitators to the Utilization of mHealth Apps to Improve Self-Care in Patients with Heart Failure | Madujibeya 2020 | Abstract |
| "In my age, we didn't have the computers": Using a complexity lens to understand uptake of diabetes eHealth innovations into primary care-A qualitative study. | Yu 2021 | Wrong patient population |
| "InTouch" with seniors: Exploring adoption of a simplified interface for social communication and related socioemotional outcomes | Judges 2017 | barriers/facilitators not explored in a meaningful way |
| "Willing but unwilling": attitudinal barriers to adoption of home-based health information technology among older adults | Young 2014 | Wrong patient population |
| 4 ACCEPTABILITY OF TELEHEALTH BY ELDERLY PATIENTS. | Odeh 2014 | Wrong study design |
| 49 Health and Social Care Professionals' Perceptions Regarding Home-Care Robots for Older People in Ireland: A Questionnaire Study...67th Annual & Scientific Meeting of the Irish Gerontological Society, Innovation, Advances and Excellence in Ageing, 26â€“28 | Kodate 2019 | Abstract |
| 62 Engaging Older Adults in Co-Creating a Virtual Coaching Assistant (CAPTAIN) to Support Independent Living at Home...67th Annual & Scientific Meeting of the Irish Gerontological Society, Innovation, Advances and Excellence in Ageing, 26â€“28 September 201 | Carroll 2019 | Abstract |
| 772 Wearable Cardiac Technologies for Older Adults, Clinician Perspectives on the Design and Application | Ferguson 2020 | barriers/facilitators not explored in a meaningful way |
| A cloud robotics solution to improve social assistive robots for active and healthy aging. | Bonaccorsi 2016 | barriers/facilitators not explored in a meaningful way |
| A comprehensive survey of wearable and wireless ECG monitoring systems for older adults. | Baig 2013 | Wrong study design |
| A dementia care training using mobile e-learning with mentoring support for home care workers: a controlled study | Su 2021 | Wrong study design |
| A Digital Coach Promoting Healthy Aging among Older Adults in Transition to Retirement: Results from a Qualitative Study in Italy | Santini 2020 | Wrong patient population |
| A Digital Human for Delivering a Remote Loneliness and Stress Intervention to At-Risk Younger and Older Adults During the covid-19 Pandemic: Randomized Pilot Trial. | Loveys 2021 | Wrong setting |
| A Digital Platform to Support Self-management of Multiple Chronic Conditions (ProACT): Findings in Relation to Engagement During a One-Year Proof-of-Concept Trial. | Doyle 2021 | Wrong patient population |
| A Feasibility Study of Multi-Component Fall Prevention for Homebound Older Adults Facilitated by Lay Coaches and Using a Tablet-Based, Gamified Exercise Application. | Choi 2021 | technology does not support care |
| A Focus Group Study on the Design Considerations and Impressions of a Socially Assistive Robot for Long-Term Care | IEEE 2014 | barriers/facilitators not explored in a meaningful way |
| A framework for evaluating mHealth tools for Older Patients on Usability. | Wildenbos 2015 | Wrong study design |
| A Game of Wants and Needs The Playful, User-centered Assessment of AAL Technology Acceptance | Schomakers 2018 | barriers/facilitators not explored in a meaningful way |
| A multi-perspective evaluation of a service robot for seniors: the voice of different stakeholders | Bedaf 2018 | barriers/facilitators not explored in a meaningful way |
| A New Governance Model for Delivering Digital Policy Agendas: A Case Study of Digital Inclusion Amongst Elderly People in the UK | Hepburn 2018 | barriers/facilitators not explored in a meaningful way |
| A Newly Developed Exergame-Based Telerehabilitation System for Older Adults: Usability and Technology Acceptance Study. | Seinsche 2023 | barriers/facilitators not explored in a meaningful way |
| A Newly Developed Web-Based Resource on Genetic Eye Disorders for Users with Visual Impairment (Gene.Vision): Usability Study | Yeong 2021 | Wrong patient population |
| A Novel Instrument for Measuring Older People's Attitudes Toward Technology (TechPH): Development and Validation. | Anderberg 2019 | barriers/facilitators not explored in a meaningful way |
| A Performance Evaluation Matrix for Measuring the Life Satisfaction of Older Adults Using eHealth Wearables. | Jeng 2022 | barriers/facilitators not explored in a meaningful way |
| A Pilot Program for Training Older Adults to use Telemedicine for Outpatient Geriatric Psychiatry | Kim 2021 | barriers/facilitators not explored in a meaningful way |
| A rapid mixed-methods evaluation of remote home monitoring models during the covid-19 pandemic in England. | Fulop 2023 | Wrong setting |
| A research on the effects of successful aging on the acceptance and use of technology of the elderly | | barriers/facilitators not explored in a meaningful way |
| A Smart Chair Physiotherapy Exergame for Fall Prevention - User Experience Study | Merilampi 2019 | barriers/facilitators not explored in a meaningful way |
| A socio-technical systems approach to the use of health IT for patient reported outcomes: Patient and healthcare provider perspectives | Wesley 2019 | barriers/facilitators not explored in a meaningful way |
| A survey analysis of telehealth access to geriatric patients during covid-19 | Mistry 2021 | barriers/facilitators not explored in a meaningful way |
| A Survey of Robots in Healthcare | Kyrarini 2021 | Wrong study design |
| A telehealth prehabilitation-to-rehabilitation intervention for older cancer surgery patients and their family caregivers | Sun 2019 | barriers/facilitators not explored in a meaningful way |
| Ability and willingness to utilize telemedicine among rheumatology patients-a cross-sectional survey. | Kong 2021 | Wrong study design |
| Acceptability and usability of a telepresence robot for geriatric primary care: A pilot | Vermeersch 2015 | barriers/facilitators not explored in a meaningful way |
| Acceptability of mHealth augmentation of Collaborative Care: A mixed methods pilot study. | Bauer 2018 | Wrong patient population |
| Acceptance and long -ter use of a social robot by elderly users in a do environment | Piasek 2018 | barriers/facilitators not explored in a meaningful way |
| Acceptance and Preferences of Using Ambient Sensor-Based Lifelogging Technologies in Home Environments. | Offermann 2021 | Wrong study design |
| Acceptance and use of health information technology by community-dwelling elders. | Fischer 2014 | Wrong study design |
| Acceptance of an assistive robot in older adults: A mixed-method study of human-robot interaction over a 1-month period in the living lab setting | Wu 2014 | Wrong setting |
| Acceptance of Cloud-Based Healthcare Services by Elderly Taiwanese People | Ku 2016 | barriers/facilitators not explored in a meaningful way |
| Acceptance of home-based telehealth problem-solving therapy for depressed, low-income homebound older adults: Qualitative interviews with the participants and aging-service case managers. | Choi 2014 | Wrong patient population |
| Acceptance of social assistive robots to support older adults with cognitive impairment and their caregivers | Pino 2013 | Abstract |
| Acceptance of Tele-Rehabilitation by Stroke Patients: Perceived Barriers and Facilitators. | Tyagi 2018 | Wrong setting |
| Acceptance of virtual consultations among older adults and caregivers in Malaysia: a pilot study during the covid-19 pandemic. | Tan 2022 | Wrong setting |
| Access and engagement disparities in digital health in cancer care: Stakeholder views on age and other contributing factors | Kemp 2019 | Wrong patient population |
| Access to smart devices and utilization of online health resources among older cardiac rehabilitation participants | Williams 2020 | Wrong patient population |
| Activity Tracker and Elderly Usability and Motivation of Mobile Healthcare in the context of Elderly People | Rasche 2015 | Wrong setting |
| Addressing telehealth barriers among older adults during covid-19 | Jezewski 2021 | Abstract |
| Adequacy of Web-Based Activities as a Substitute for In-Person Activities for Older Persons During the covid-19 Pandemic: Survey Study. | Cohen-Mansfield 2021 | Wrong setting |
| Adoption of online health management tools among healthy older adults: An exploratory study. | Zettel-Watson 2016 | Wrong patient population |
| Advancing Telemedicine Services for the Aging Population: The challenge of Interoperability | vanVelsen 2015 | barriers/facilitators not explored in a meaningful way |
| Aesthetically Designing Video-Call Technology With Care Home Residents: A Focus Group Study | Zamir 2021 | barriers/facilitators not explored in a meaningful way |
| Affective Technology for Older Adults: Does Fun Technology Affect Older Adults and Change Their Lives? | Fukuda 2011 | barriers/facilitators not explored in a meaningful way |
| An app for supporting older people receiving home care - usage, aspects of health and health literacy: a quasi-experimental study. | GÃ¶ransson 2020 | barriers/facilitators not explored in a meaningful way |
| AN END-USER PERSPECTIVE ON THE CAMI AMBIENT AND ASSISTED LIVING PROJECT | Awada 2018 | Abstract |
| An ethnographical study of the accessibility barriers in the everyday interactions of older people with the web | Sayago 2011 | Wrong patient population |
| An Innovative Technology to Support Independent Living: he Smarter Safer Homes Platform...5th Global Telehealth meeting, Adelaide, Australia, November 2017. | KARUNANITHI 2018 | Abstract |
| An Online Dual-Task Cognitive and Motor Exercise Program for Individuals With Parkinson Disease (PD3 Move Program): Acceptability Study. | Domingos 2022 | Wrong study design |
| Analyzing older users' home telehealth services acceptance behavior-applying an Extended UTAUT model. | Cimperman 2016 | barriers/facilitators not explored in a meaningful way |
| Apple HealthKit and Health App: Patient Uptake and Barriers in Primary Care | North 2016 | Wrong patient population |
| Application of persuasive systems design principles to design a self-management application user interface for Hispanic informal dementia caregivers: User preferences and perceptions | Lucero 2022 | Wrong study design |
| Are the Elderly Averse to Technology? | Jones 2020 | barriers/facilitators not explored in a meaningful way |
| Assembling mass-market technology for the sake of wellbeing: a case study on the adoption of ambient intelligent systems by older adults living at home | Gutierrez 2019 | barriers/facilitators not explored in a meaningful way |
| Assessing Older Adults' Perceptions of Sensor Data and Designing Visual Displays for Ambient Environments | Reeder 2014 | barriers/facilitators not explored in a meaningful way |
| Assessment of the Elderly on Perceived Needs, Benefits and Barriers: Inputs for the Design of Intelligent Assistive Technology | Albina 2018 | Abstract |
| Assistive technologies after stroke: self-management or fending for yourself? A focus group study | Demain 2013 | Wrong patient population |
| Attitude and perceptions of older and younger adults towards ambient technology for assisted living | Choukou 2021 | Wrong patient population |
| Attitudes and Perceptions Toward Healthcare Technology Adoption Among Older Adults in Singapore: A Qualitative Study | Low 2021 | Wrong patient population |
| Attitudes and Preferences on the Use of Mobile Health Technology and Health Games for Self-Management: Interviews With Older Adults on Anticoagulation Therapy | Lee 2014 | barriers/facilitators not explored in a meaningful way |
| Attitudes of elderly Austrians towards new technologies: communication and entertainment versus health and support use | Halmdienst 2019 | barriers/facilitators not explored in a meaningful way |
| Attitudes of Health Care Professionals Toward Older Adults' Abilities to Use Digital Technology: Questionnaire Study. | Mannheim 2021 | barriers/facilitators not explored in a meaningful way |
| Augmented video consultations in care homes during the covid-19 pandemic: a qualitative study. | Ochieing 2022 | Wrong setting |
| Awareness and attitudes of elderly Southeast Asian adults towards telehealth during the covid-19 pandemic: a qualitative study | Man 2023 | Wrong setting |
| Baby boomers' adoption of consumer health technologies: survey on readiness and barriers | LeRouge 2014 | Wrong patient population |
| Barriers and enablers for older adults participating in a home-based pragmatic exercise program delivered and monitored by Amazon Alexa: a qualitative study. | Jansons 2022 | technology does not support care |
| Barriers and Facilitators in Implementing Non-Face-to-Face Chronic Care Management in an Elderly Population with Diabetes: A Qualitative Study of Physician and Health System Perspectives | Bazzano 2018 | Wrong patient population |
| Barriers and facilitators of older people's mHealth usage: A qualitative review of older people's views. | Spann 2018 | Wrong study design |
| Barriers and facilitators to mobile health and active surveillance use among older adults with skin disease | Johnson 2021 | Wrong patient population |
| Barriers and facilitators to telemedicine: Can you hear me now? | Lopez 2021 | Wrong study design |
| Barriers and Facilitators to the Adoption of Mobile Health Among Health Care Professionals From the United Kingdom: Discrete Choice Experiment | Leigh 2020 | Wrong patient population |
| Barriers and facilitators to using the CommFitâ„¢ smart phone app to measure talk time for people with aphasia. | Brandenburg 2017 | barriers/facilitators not explored in a meaningful way |
| Barriers and facilitators to virtual care in a geriatric medicine clinic: a semi-structured interview study of patient, caregiver and healthcare provider perspectives. | Watt 2022 | Wrong setting |
| Barriers in acceptance of Ambient Assisted Living Technologies among Older Australians | IEEE 2017 | Abstract |
| Barriers in telehealth access in senior living communities | Mao 2021 | Abstract |
| BARRIERS TO ACCEPTANCE AND LEARNING OF ICT TECHNOLOGIES IN AN EUROPEAN AMBIENT ASSISTED LIVING PROJECT | Cramariuc 2017 | Wrong study design |
| Barriers to and Facilitators of Engagement With mHealth Technology for Remote Measurement and Management of Depression: Qualitative Analysis. | Simblett 2019 | Wrong patient population |
| Barriers to patient participation in a self-management and education website Renal PatientView: A questionnaire-based study of inactive users | Hazara 2016 | barriers/facilitators not explored in a meaningful way |
| Barriers to Patient Portal Access and Use: Evidence from the Health Information National Trends Survey | El-Toukhy 2020 | barriers/facilitators not explored in a meaningful way |
| Barriers to technology use among older heart failure individuals in managing their symptoms after hospital discharge | Nguyen 2017 | Wrong patient population |
| Barriers to telehealth access among homebound older adults | Kalicki 2021 | Wrong study design |
| Barriers to telehealth: The patient perspective | Bodine 2020 | Wrong study design |
| Behavioral Mechanisms Of A Sensor-Controlled Digital Game That Motivates Self-Management Behaviors By Older Adults With Heart Failure | DeMain 2022 | technology does not support care |
| Benefits and Barriers of Technology for Home Function and Mobility Assessment: Perspectives of Older Patients With Blood Cancers, Caregivers, and Clinicians. | Clancey 2023 | Wrong patient population |
| Benefits and Challenges of Diabetes Technology Use in Older Adults | Toschi 2020 | Wrong study design |
| Breaking Digital Barriers: A Social-Cognitive Approach to Improving Digital Literacy in Older Adults | Steelman 2016 | Wrong study design |
| Bridging the User Barriers of Home Telecare | Woll 2020 | barriers/facilitators not explored in a meaningful way |
| Can a Free Wearable Activity Tracker Change Behavior? The Impact of Trackers on Adults in a Physician-Led Wellness Group | Gualtieri 2016 | Wrong patient population |
| Can a service robot which supports independent living of older people disobey a command? The views of older people, informal carers and professional caregivers on the acceptability of robots. | Bedaf 2016 | barriers/facilitators not explored in a meaningful way |
| Can an Intelligent Virtual Assistant (IVA) Meet Older Adult Health-Related Needs in the Context of a Geriatric 5Ms Framework? | Lifset 2022 | Abstract |
| Canadian Occupational Therapists' Use of Technology With Older Adults: A Nationwide Survey. | Aboujaoude 2021 | Wrong study design |
| Cancer survivors' receptiveness to digital technology-supported physical rehabilitation and the implications for design: Qualitative study | Christensen 2020 | barriers/facilitators not explored in a meaningful way |
| Care managers' perceptions of eHomecare: a qualitative interview study. | Ã…kerlind 2019 | barriers/facilitators not explored in a meaningful way |
| Care robot orientation: What, who and how? Potential users' perceptions. | Johansson-Pajala 2020 | barriers/facilitators not explored in a meaningful way |
| Caregivers' experiences with the selection and use of assistive technology | Pysklywec 2018 | barriers/facilitators not explored in a meaningful way |
| Caregivers' Technology Acceptance of an In-Home Care Management App: A Mixed-Methods Study. | Wen 2022 | barriers/facilitators not explored in a meaningful way |
| Carers' involvement in telecare provision by local councils for older people in England: perspectives of council telecare managers and stakeholders. | Steils 2021 | barriers/facilitators not explored in a meaningful way |
| Carer's perception on social assistive technology acceptance and adoption: moderating effects of perceived risks. | Khaksar 2021 | barriers/facilitators not explored in a meaningful way |
| Caring by telecare? A hermeneutic study of experiences among older adults and their family caregivers. | Karlsen 2019 | barriers/facilitators not explored in a meaningful way |
| Caring callers: the impact of the telephone reassurance program on homebound older adults during covid-19. | Lee 2021 | technology does not support care |
| Challenges in using wearable GPS devices in low-income older adults: Can map-based interviews help with assessments of mobility? | Schmidt 2019 | barriers/facilitators not explored in a meaningful way |
| Changes in technology acceptance among older people with dementia: the role of social robot engagement. | Ke 2020 | barriers/facilitators not explored in a meaningful way |
| Clinicians' perspectives on a Web-based system for routine outcome monitoring in old-age psychiatry in the Netherlands | Veerbeek 2012 | barriers/facilitators not explored in a meaningful way |
| Co-construction of an Internet-based intervention for older assistive technology users and their family caregivers: stakeholders' perceptions. | GÃ©linas-Bronsard 2019 | barriers/facilitators not explored in a meaningful way |
| Co-creation of mHealth intervention for older adults with hip fracture and family caregivers: a qualitative study | Ariza-Vega 2022 | Wrong patient population |
| Comparison of Mobile Health Technology Use for Self-Tracking Between Older Adults and the General Adult Population in Canada: Cross-Sectional Survey | Jaana 2020 | barriers/facilitators not explored in a meaningful way |
| Comprehensive Senior Technology Acceptance Model of Daily Living Assistive Technology for Older Adults With Frailty: Cross-sectional Study. | Shin 2023 | Wrong study design |
| Conditions and ethical challenges that could influence the implementation of technologies in nursing homes: A qualitative study. | Bourbonnais 2019 | barriers/facilitators not explored in a meaningful way |
| covid-19's Influence on Information and Communication Technologies in Long-Term Care: Results From a Web-Based Survey With Long-Term Care Administrators. | Schuster 2022 | Wrong study design |
| Dances with Social Robots: A Pilot Study at Long-Term Care | Li 2022 | barriers/facilitators not explored in a meaningful way |
| Decision-making Factors Associated With Telehealth Adoption by Patients With Heart Failure at Home: A Qualitative Study | Woo 2020 | barriers/facilitators not explored in a meaningful way |
| Delivery of Home-Based Exercise Interventions in Older Adults Facilitated by Amazon Alexa: A 12-week Feasibility Trial. | Jansons 2022 | Wrong patient population |
| Dementia Caregiver Insights on Use of Assistive Technologies | Mikula 2022 | Wrong setting |
| Design Considerations for Mobile Health Applications Targeting Older Adults | Li 2021 | Wrong study design |
| Design of the user interface for "Stappy", a sensor-feedback system to facilitate walking in people after stroke: a user-centred approach | Braun 2020 | barriers/facilitators not explored in a meaningful way |
| Designing an Online Social Support Platform Through Co-Creation with Seniors..."Building Continents of Knowledge in Oceans of Data: The Future of Co-Created eHealth," EFMI, Medical Informatics Europe (MIE), April 24-26th, 2018, Gothenburg, Sweden. | ROCHAT 2018 | Wrong patient population |
| Designing and evaluating an electronic patient falls reporting system: Perspectives for the implementation of health information technology in long-term residential care facilities | Mei 2013 | barriers/facilitators not explored in a meaningful way |
| Designing Service Robots for Senior Citizens: Exploring the Requirement Space | DEStechPublicatInc 2016 | barriers/facilitators not explored in a meaningful way |
| Determinants of and Willingness to Use and Pay for Digital Health Technologies Among the Urban Elderly in Hangzhou, China | Yang 2023 | barriers/facilitators not explored in a meaningful way |
| Determinants of information communication and smart home automation technology adoption for aging-in-place | Arthanat 2020 | barriers/facilitators not explored in a meaningful way |
| Determinants of intention with remote health management service among urban older adults: A Unified Theory of Acceptance and Use of Technology perspective. | Li 2023 | barriers/facilitators not explored in a meaningful way |
| Developing a pragmatic evaluation of ICTs for older adults with cognitive impairment at scale: the IN LIFE experience | Astell 2022 | Wrong study design |
| Developing a questionnaire to understand perceptions towards home-care robots among older people who receive home-care, family caregivers, and home-care professionals | Tsujimura 2017 | Abstract |
| Development and Evaluation of the Usefulness, Usability, and Feasibility of iNNOV Breast Cancer: Mixed Methods Study | Mendes-Santos 2022 | Wrong patient population |
| DEVELOPMENT AND PILOT TESTING OF AN ARTIFICIAL INTELLIGENCE-BASED FAMILY CAREGIVER NEGOTIATION TRAINING PROGRAM: NEGOTIAGE | Murawski 2023 | Wrong study design |
| Development of a Healthcare Information System for Community Care of Older Adults and Evaluation of Its Acceptance and Usability. | Choi 2022 | barriers/facilitators not explored in a meaningful way |
| Development of an Evaluative, Educational, and Communication-Facilitating App for Older Adults with Chronic Low Back Pain: Patient Perceptions of Usability and Utility. | Madill 2019 | barriers/facilitators not explored in a meaningful way |
| Development of opportunities to provide medication treatment for seniors through mobile applications | Babaskin 2023 | barriers/facilitators not explored in a meaningful way |
| Dialogue cafes with older adults as method for appraising prioritization of technological solutions | Halvorsrud 2019 | Wrong study design |
| Differences in Access to and Preferences for Using Patient Portals and Other eHealth Technologies Based on Race, Ethnicity, and Age: A Database and Survey Study of Seniors in a Large Health Plan | Gordon 2016 | Wrong study design |
| DigiSwitch: A Device to Allow Older Adults to Monitor and Direct the Collection and Transmission of Health Information Collected at Home | Caine 2011 | barriers/facilitators not explored in a meaningful way |
| DIGITAL ASSISTIVE TECHNOLOGIES FOR AGEING PEOPLE - LEARNING BARRIERS AND EDUCATIONAL APPROACHES | Helander 2019 | Wrong patient population |
| Digital health care for older adults. | Evangelista 2019 | barriers/facilitators not explored in a meaningful way |
| Digital personal assistants are smart ways for assistive technology to aid the health and wellbeing of patients and carers. | Balasubramanian 2021 | barriers/facilitators not explored in a meaningful way |
| Digital profiling and analysis of barriers to teleconsultations among ophthalmic patients in North West London (NWL) | Mushtaq 2021 | Wrong patient population |
| DIGITAL VOICE ASSISTANTS: A NOVEL TECHNOLOGICAL APPROACH TO SUPPORTING OLDER ADULTS TO SELF-MANAGE MUSCULOSKELETAL CONDITIONS | Scott 2023 | Abstract |
| Does telemonitoring in heart failure empower patients for self-care? A qualitative study | Riley 2013 | barriers/facilitators not explored in a meaningful way |
| Does the purpose matter? A comparison of everyday information and communication technologies between eHealth use and general use as perceived by older adults with cognitive impairment | Jakobsson 2020 | barriers/facilitators not explored in a meaningful way |
| Dutch nurses' willingness to use home telehealth: implications for practice and education | vanHouwelingen 2015 | Wrong patient population |
| Effectiveness of a tablet-based intervention for people living with dementia in primary care-A cluster randomized controlled trial | Lech 2023 | Wrong study design |
| Effects of Mobile App-Based Intervention for Depression in Middle-Aged and Older Adults: Mixed Methods Feasibility Study. | Gould 2021 | Wrong patient population |
| eHealth Interventions for Dementia - Using WordPress Plugins as a Flexible Dissemination for Dementia Service Providers. | REICHOLD 2021 | barriers/facilitators not explored in a meaningful way |
| Embracing digital technology in response to the covid - 19 pandemic: A patient satisfaction service evaluation | Hadley-Barrows 2022 | Wrong patient population |
| Emotional reactions of elderly people towards social assistive robots | Ruf 2022 | Not in English |
| Empowering caregivers to customizing the assistive computing support of older adults - an end-user domain-specific approach. | Giraud 2020 | barriers/facilitators not explored in a meaningful way |
| Enablers for and barriers to using My Kanta - A focus group study of older adults' perceptions of the National Electronic Health Record in Finland | Eriksson-Backa 2021 | Wrong patient population |
| Engagement and experience of older people with socially assistive robots in home care. | Khosla 2021 | barriers/facilitators not explored in a meaningful way |
| Environmental barriers to participation and facilitators for use of three types of assistive technology devices. | Widehammar 2019 | barriers/facilitators not explored in a meaningful way |
| E-social work and at-risk populations: technology and robotics in social intervention with elders. The case of Spain. | delaFuenteRobles 2019 | barriers/facilitators not explored in a meaningful way |
| Ethical concerns with the use of intelligent assistive technology: findings from a qualitative study with professional stakeholders | Wangmo 2019 | Wrong patient population |
| Ethics of socially assistive robots in aged-care settings: a socio-historical contextualisation | Vandemeulebroucke 2020 | Wrong study design |
| Evaluating user experiences of the secure messaging tool on the Veterans Affairs' patient portal system | Haun 2014 | Wrong patient population |
| Evaluation of 1-Year in-Home Monitoring Technology by Home-Dwelling Older Adults, Family Caregivers, and Nurses. | Pais 2020 | barriers/facilitators not explored in a meaningful way |
| Evaluation of an eHealth Intervention in Chronic Care for Frail Older People: Why Adherence is the First Target. | Makai 2014 | barriers/facilitators not explored in a meaningful way |
| Evaluation of the Usability and Acceptability of the InnoWell Platform as Rated by Older Adults: Survey Study. | LaMonica 2021 | Wrong study design |
| Everybody hurts sometimes: perceptions of benefits and barriers in telemedical consultations. | Rohowsky 2023 | Wrong patient population |
| Everyday technology use among older deaf adults. | Singleton 2019 | barriers/facilitators not explored in a meaningful way |
| Examining social determinants in use of assistive technology for race/ethnic groups of older adults | Chan 2020 | Wrong study design |
| Experiences of family caregivers of persons living with dementia with and without a smart- clothes assisted home nursing program during the heightened covid-19 alert | Sung 2022 | Wrong setting |
| Experiences of health and social professionals using care technologies with older adults during the covid-19 pandemic: A qualitative study. | Martin-Palomo 2024 | Wrong setting |
| Experiences of Older People and Social Inclusion in Relation to Smart "Age-Friendly" Cities: A Case Study of Chongqing, China. | Li 2021 | technology does not support care |
| Experiences with health technologies to support physical activity in people over the age of 65: A qualitative survey of the requirements for the development of preventive technologies for a heterogeneous target group | Pauls 2019 | Not in English |
| Experiences with the use of welfare technologies for elderly persons | Grut 2019 | Wrong study design |
| Exploring community members' perceptions to adopt a Tele-COPD program in rural counties | Alexander 2021a | Wrong patient population |
| Exploring influencing factors of technology use for active and healthy ageing support in older adults. | Vaziri 2020 | barriers/facilitators not explored in a meaningful way |
| Exploring Older Adults' Experiences of a Home-Based, Technology-Driven Balance Training Exercise Program Designed to Reduce Fall Risk: A Qualitative Research Study Within a Randomized Controlled Trial. | Ambrens 2023 | Wrong patient population |
| Exploring Patient and Staff Experiences With Video Consultations During covid-19 in an English Outpatient Care Setting: Secondary Data Analysis of Routinely Collected Feedback Data. | Bradwell 2022 | Wrong setting |
| Exploring the acceptability of sleep monitoring and bright light technologies for intervention use in older adults | Leggett 2015 | barriers/facilitators not explored in a meaningful way |
| Exploring the Digital Divide as a Barrier to Use of a Personal Health Record in the Elderly. | Frutos 2022 | Wrong study design |
| Exploring the Perspectives and Experiences of Older Adults With Asthma and Chronic Obstructive Pulmonary Disease Toward Mobile Health: Qualitative Study. | Kouri 2023 | Wrong patient population |
| Exploring the se of Technology for Active Aging and Thriving. | Ã˜DERUD 2017 | barriers/facilitators not explored in a meaningful way |
| Facilitating Digital Communication in Seniors | IEEE 2016 | Abstract |
| Facilitating medication adherence in elderly care using ubiquitous sensors and mobile social networks | Yu 2015 | barriers/facilitators not explored in a meaningful way |
| Facilitating telehealth for older adults during the covidâ€19 pandemic and beyond: Strategies from a Singapore geriatric center. | Tan 2020 | Wrong study design |
| Facilitators and Barriers to the Adoption of Telehealth in Older Adults. | | Wrong study design |
| Facilitators of and Barriers to mHealth Adoption in Older Adults With Heart Failure | Cajita 2018 | Wrong patient population |
| Facilitators of and barriers to the use of a computer-based self-monitoring system by type 2 diabetic and/or hypertensive patients | Yan 2023 | Wrong patient population |
| Factors affecting the acceptability of technology in health care among older korean adults with multiple chronic conditions: A cross-sectional study adopting the senior technology acceptance model | Ha 2020 | barriers/facilitators not explored in a meaningful way |
| Factors Associated With Caregivers' Preference for Digital Technologies to Support Older Adults. | Hwang 2022 | barriers/facilitators not explored in a meaningful way |
| Factors Associated with the Acceptance of New Technologies for Ageing in Place by People over 64 Years of Age. | Chimento-Diaz 2022 | Wrong study design |
| Factors Associated with Veteran Self-Reported Use of Digital Health Devices | Robinson 2024 | barriers/facilitators not explored in a meaningful way |
| Factors influencing trust in Ambient Assisted Living Technology: A scenario-based analysis. | Steinke 2014 | barriers/facilitators not explored in a meaningful way |
| Factors relating to home telehealth acceptance and usage compliance | Wade 2012 | barriers/facilitators not explored in a meaningful way |
| Faller Classification in Older Adults Using Wearable Sensors Based on Turn and Straight-Walking Accelerometer-Based Features | Drover 2017 | barriers/facilitators not explored in a meaningful way |
| Falling off the bandwagon? Exploring the challenges to sustained digital engagement by older people | Damodaran 2014 | Wrong patient population |
| Family caregiver readiness to adopt smart home technology to monitor care-Dependent older adults: A qualitative exploratory study | Dermody 2023 | Wrong patient population |
| Family caregivers' conceptions of usage of and information on products, technology and Web-based services | Edlund 2011 | barriers/facilitators not explored in a meaningful way |
| Family caregivers' experience of care and use of assistive technologies | Lettre 2019 | barriers/facilitators not explored in a meaningful way |
| Family carers' perspectives of managing activities of daily living and use of mHealth applications in dementia care: A qualitative study | Calleja 2019 | barriers/facilitators not explored in a meaningful way |
| Feasibility and acceptability of an education and training e-resource to support the sexuality, intimacy and relationship needs of older care home residents: a mixed methods study. | Horne 2022 | technology does not support care |
| Feasibility of a covid-19 Rapid Response Telehealth Group Addressing Older Adult Worry and Social Isolation. | Weiskittle 2022 | barriers/facilitators not explored in a meaningful way |
| Feasibility of a wireless health monitoring system for prevention and health assessment of elderly people | Jimenez-Mixco 2013 | barriers/facilitators not explored in a meaningful way |
| Feasibility of an Automated Bidet Intervention to Decrease Caregiver Burden. | Bollinger 2021 | barriers/facilitators not explored in a meaningful way |
| Findings From Talking Tech: A Technology Training Pilot Intervention to Reduce Loneliness and Social Isolation Among Homebound Older Adults | Gadbois 2022 | Wrong patient population |
| Formations for Facilitating Communication Among Robotic Wheelchair Users and Companions | Kobayashi 2015 | barriers/facilitators not explored in a meaningful way |
| From 65 to 103, Older Adults Experience Virtual Reality Differently Depending on Their Age: Evidence from a Large-Scale Field Study in Nursing Homes and Assisted Living Facilities. | Moore 2023 | barriers/facilitators not explored in a meaningful way |
| Geriatric Remote Initiative (GeRI): Qualitative Feedback from Older Cancer Survivors and Caregivers About Home-Based Technology for Assessing Symptoms and Function During Cancer Care | Mir 2023 | Wrong patient population |
| Going Remote-Demonstration and Evaluation of Remote Technology Delivery and Usability Assessment With Older Adults: Survey Study | Hill 2021 | barriers/facilitators not explored in a meaningful way |
| Habit Formation in Wearable Activity Tracker Use Among Older Adults: Qualitative Study. | Peng 2021 | barriers/facilitators not explored in a meaningful way |
| Health Access, Health Promotion, and Health Self-Management: Barriers When Building Comprehensive Ageing Communities. | Perez-Saiz 2023 | barriers/facilitators not explored in a meaningful way |
| Health and self-perceived barriers to internet use among older migrants: a population-based study. | Kouvonen 2022 | Wrong study design |
| Health Care Personnel's Perspective on Potential Electronic Health Interventions to Prevent Hospitalizations for Older Persons Receiving Community Care: Qualitative Study. | Gjestsen 2020 | barriers/facilitators not explored in a meaningful way |
| Health education during a pandemic: An online intervention about the responsible use of medicines in older people | Cano 2023 | Wrong setting |
| Health literacy, digital health literacy and the implementation of digital health technologies in cancer care: the need for a strategic approach. | | Wrong patient population |
| Health monitoring through wearable technologies for older adults: Smart wearables acceptance model | Li 2019 | Wrong study design |
| Health professionals' and researchers' views on Intelligent Assistive Technology for psychogeriatric care. | lenca 2018 | barriers/facilitators not explored in a meaningful way |
| HEALTH PROFESSIONALS' USER EXPERIENCE OF THE INTELLIGENT BED IN PATIENTS' HOMES. | Cai 2015 | barriers/facilitators not explored in a meaningful way |
| Health workers' perceptions and experiences of using mHealth technologies to deliver primary healthcare services: a qualitative evidence synthesis | Odendaal 2020 | Wrong study design |
| Health workers' perceptions and experiences of using mHealth technologies to deliver primary healthcare services: a qualitative evidence synthesis. | Odendaal 2020 | Wrong study design |
| Healthcare Provider Perspectives on Digital and Interprofessional Medication Management in Chronically Ill Older Adults of Turkish Descent in Germany: A Qualitative Structuring Content Analysis. | Bird 2022 | Wrong patient population |
| Home telehealth: facilitators, barriers, and impact of nurse support among high-risk dialysis patients | Minatodani 2013 | Wrong patient population |
| Home Visit with Telemedicine Support after Emergency Department Discharge to Promote Age Friendly Care | McQouwn 2023 | Wrong study design |
| Homebound patients' perspectives on technology and telemedicine: A qualitative analysis | Huang 2016 | Wrong patient population |
| Home-care robots - Attitudes and perceptions among older people, carers and care professionals in Ireland: A questionnaire study | Donnelly 2021 | barriers/facilitators not explored in a meaningful way |
| How older adults make decisions regarding smart technology: an ethnographic approach | Davenport 2012 | barriers/facilitators not explored in a meaningful way |
| Human factors/usability barriers to home medical devices among individuals with disabling conditions: in-depth interviews with positive airway pressure device users. | Fung 2015 | Wrong patient population |
| ICT Inexperienced Elderlies: What Would Attract Elderlies to Use Items of Technology? | Koscher 2017 | Wrong study design |
| Identification of Factors Influencing the Adoption of Health Information Technology by Nurses Who Are Digitally Lagging: In-Depth Interview Study | DeLeeuw 2020 | Wrong patient population |
| Identifying features that enhance older adults' acceptance of robots: A mixed methods study | Chen 2019 | Wrong patient population |
| Identifying Preferred Appearance and Functional Requirements of Aged Care Robots Among Older Chinese Immigrants: Cross-Sectional Study. | Chiu 2023 | barriers/facilitators not explored in a meaningful way |
| Implementation of a Statewide Web-Based Caregiver Resource Information System (CareNav): Mixed Methods Study. | Young 2022 | Wrong setting |
| Implementation of Telemonitoring in Health Care: Facilitators and Barriers for Using eHealth for Older Adults with Chronic Conditions. | Liljeroos 2023 | Wrong patient population |
| Implementing a Telehealth Support Tool for Community-Dwelling Older Adults During the covid-19 Pandemic: A Qualitative Investigation of Provider Experiences | Dassieu 2022 | Wrong setting |
| Improving a Web-Based Tool to Support Older Adults to Stay Independent at Home: Qualitative Study | Garvelink 2020 | barriers/facilitators not explored in a meaningful way |
| Improving uptake and outcomes of hearing aid fitting for older adults: What are the barriers and facilitators? | Hickson 2014 | Wrong patient population |
| Improvised use of a digital tool for social interaction in a Norwegian care facility during the covid-19 pandemic: an exploratory study. | Badawy 2022 | barriers/facilitators not explored in a meaningful way |
| Increasing Caregiver Access to Programming: A Qualitative Exploration of Caregivers' Experience of a Telehealth Powerful Tools for Caregivers Program | Serwe 2019 | barriers/facilitators not explored in a meaningful way |
| Increasing the acceptance of assistive robots for older people through marketing strategies based on stakeholder needs. | Glende 2016 | barriers/facilitators not explored in a meaningful way |
| Informal dementia caregivers: Current technology use and acceptance of technology in care | Rydlewska-Liszkowska 2021 | barriers/facilitators not explored in a meaningful way |
| In-Home Monitoring Technologies: Perspectives and Priorities of Older Adults | Wild 2012 | Wrong study design |
| Integrating Artificial Intelligence and Wearable IoT System in Long-Term Care Environments. | Wang 2023 | barriers/facilitators not explored in a meaningful way |
| Internet-of-Things Smart Home Technology to Support Aging-in-Place: Older Adults' Perceptions and Attitudes | Choi 2021 | barriers/facilitators not explored in a meaningful way |
| Interviews with family caregivers of older adults: Their experiences of care and the integration of assistive technology in care | Beaudoin 2020 | barriers/facilitators not explored in a meaningful way |
| IoT/Sensor-Based Infrastructures Promoting a Sense of Home, Independent Living, Comfort and Wellness | Cahill 2019 | barriers/facilitators not explored in a meaningful way |
| iPad Use Among Older Women with Low Vision: Follow-Up Focus Group Findings. | Smallfield 2023 | Wrong study design |
| Legal Aspects on Smart House Welfare Technology for Older People in Norway | Sanchez 2016 | Wrong study design |
| Lessons from covid-19 pandemic: Clinical experiences on telemedicine in patients with dementia in Iran | Noroozian 2021 | Wrong study design |
| Lessons Learned From an Effectiveness Evaluation of Inlife, a Web-Based Social Support Intervention for Caregivers of People With Dementia: Randomized Controlled Trial | Christie 2022 | barriers/facilitators not explored in a meaningful way |
| Lessons learned: feasibility and acceptability of a telehealth-delivered exercise intervention for rurald-welling individuals with dementia and their caregivers | DalBello-Haas 2014 | barriers/facilitators not explored in a meaningful way |
| Let us Meet Online! Examining the Factors Influencing Older Chinese's Social Networking Site Use | Zhou 2019 | Wrong patient population |
| Living with a Mobile Companion Robot in your Own Apartment - Final Implementation and Results of a 20-Weeks Field Study with 20 Seniors | IEEE 2019 | barriers/facilitators not explored in a meaningful way |
| Managers' and Administrators' Perspectives on Digital Technology Use in Regional Long-Term Care Homes During the covid-19 Pandemic. | Kowaja 2023 | Wrong setting |
| Managing Multimorbidity: Identifying Design Requirements for a Digital Self-Management Tool to Support Older Adults with Multiple Chronic Conditions | AssocCompMachinery 2019 | barriers/facilitators not explored in a meaningful way |
| Maximising the value of touchscreen tablet devices for people living with dementia | Joddrell 2016 | barriers/facilitators not explored in a meaningful way |
| Maximizing Telerehabilitation for Patients with Visual Loss after Stroke: Interview and Focus Group Study with Stroke Survivors, Carers, and Occupational Therapists | Dunne 2020 | barriers/facilitators not explored in a meaningful way |
| Mending the Cracks: A Case Study in Using Technology to Assist with Transitional Care for Persons with Dementia | Ritchie 2019 | barriers/facilitators not explored in a meaningful way |
| Mixed feelings: general practitioners' attitudes towards eHealth for stress urinary incontinence - a qualitative study. | Firet 2019 | Wrong patient population |
| Mobile health for older adult patients: Using an aging barriers framework to classify usability problems. | Wildenbos 2019 | Wrong patient population |
| Modelling factors influencing the adoption of smart-home technologies | Wong 2016 | barriers/facilitators not explored in a meaningful way |
| Moderating factors influencing adoption of a mobile chronic disease management system in China. | Zhu 2018 | Wrong patient population |
| Motivators and barriers to using information and communication technology in everyday life following stroke: A qualitative and video observation study. | Lemke 2020 | Wrong patient population |
| Moving beyond 'safety' versus 'autonomy': a qualitative exploration of the ethics of using monitoring technologies in long-term dementia care | Hall 2019 | barriers/facilitators not explored in a meaningful way |
| NegotiAge: Pilot Testing an Artificial Intelligence-Based Family Caregiver Negotiation Training Program | Murawski 2023 | Abstract |
| New musical interfaces for older adults in residential care: assessing a user-centred design approach. | Taylor 2023 | barriers/facilitators not explored in a meaningful way |
| Nothing Else Matters! Trade-Offs Between Perceived Benefits and Barriers of AAL Technology Usage | Offermann-vanHeek 2019 | Wrong patient population |
| Novel mHealth App to Deliver Geriatric Assessment-Driven Interventions for Older Adults With Cancer: Pilot Feasibility and Usability Study | Loh 2018 | Wrong patient population |
| Novel telemedicine technologies in geriatric chronic non-cancer pain: Primary care providers' perspectives | Levine 2014 | Wrong patient population |
| Nursing home staff members' attitudes and knowledge about urinary incontinence: the impact of technology and training | Ehlman 2012 | barriers/facilitators not explored in a meaningful way |
| Nursing staff's evaluation of facilitators and barriers during implementation of wireless nurse call systems in residential care facilities. A cross-sectional study | Dugstad 2020 | Wrong study design |
| Old-age diversity is underrepresented in digital health research: findings from the evaluation of a mobile phone system for post-operative progress monitoring in Sweden. | Poli 2023 | Wrong study design |
| Older Adults' and Family Caregivers' Technological Arrangements on Risk of Institutionalization | Kim 2023 | Wrong study design |
| Older Adults' and Family Caregivers' Technological Arrangements on Risk of Institutionalization. | Kim 2024 | Wrong study design |
| Older adults are mobile too!Identifying the barriers and facilitators to older adults' use of mHealth for pain management. | Parker 2013 | Duplicate |
| Older adults' attitudes and barriers toward the use of mobile phones | Navabi 2016 | Wrong study design |
| Older Adults' Engagement in Technologyâ€Mediated Selfâ€Monitoring of Diet: A Mixedâ€Method Study. | Aure 2021 | barriers/facilitators not explored in a meaningful way |
| Older adults' experiences and perceptions of digital technology: (Dis)empowerment, wellbeing, and inclusion | Hill 2015 | barriers/facilitators not explored in a meaningful way |
| Older Adults' Experiences with Audiovisual Virtual Reality: Perceived Usefulness and Other Factors Influencing Technology Acceptance. | Roberts 2019 | barriers/facilitators not explored in a meaningful way |
| Older Adults' Perceptions of ICT: Main Findings from the Technology In Later Life (TILL) Study | Marston 2019 | Wrong patient population |
| Older Adults' Perceptions of the Usefulness of Technologies for Engaging in Physical Activity: Using Focus Groups to Explore Physical Literacy. | Campelo 2020 | barriers/facilitators not explored in a meaningful way |
| Older Adults' Perspectives on Using Digital Technology to Maintain Good Mental Health: Interactive Group Study | Andrews 2019 | Wrong patient population |
| Older adults' use of an online decision support system: Usability and stability of assistive technology recommendations. | Auger 2022 | barriers/facilitators not explored in a meaningful way |
| Older Adults' Use of and Interest in Technology and Applications for Health Management: A Survey Study. | Sproul 2023 | Wrong study design |
| Older Adults' Use of Technology. | Wallace 2013 | Wrong study design |
| Older family carers in rural areas: experiences from using caregiver support services based on Information and Communication Technology (ICT). | Blusi 2013 | barriers/facilitators not explored in a meaningful way |
| Older people and rural eHealth: perceptions of caring relations and their effects on engagement in digital primary health care | Lindberg 2021 | barriers/facilitators not explored in a meaningful way |
| Older people and technology: Time to smarten up our act | Ahmed 2021 | Wrong study design |
| Older People Negotiating Independence and Safety in Everyday Life Using Technology: Qualitative Study. | Stokke 2018 | barriers/facilitators not explored in a meaningful way |
| On the Use of Assistive Technology during the covid-19 Outbreak: Results and Lessons Learned from Pilot Studies. | Fiorini 2022 | barriers/facilitators not explored in a meaningful way |
| ONE YEAR OF TELEMEDICINE IN THE MANAGEMENT OF GENITOURINARY MALIGNANCIES | Margolin 2022 | Abstract |
| Online Patient Portal Use by Older Adults in Rural Kentucky. | Jones 2017 | Abstract |
| Opportunities and challenges for self-monitoring technologies for healthy aging: An in-situ study. | Randriambelonoro 2017 | barriers/facilitators not explored in a meaningful way |
| Overcoming the Digital Divide for Older Patients With Respiratory Disease: Focus Group Study. | Metting 2023 | Wrong patient population |
| Participative development of a new technological tool for supervision of older adults in nursing home: The e-monitor'age project example | Fumel 2015 | barriers/facilitators not explored in a meaningful way |
| Patient Acceptability of Home Monitoring for Neovascular Age-Related Macular Degeneration Reactivation: A Qualitative Study. | O'Connor 2022 | Wrong patient population |
| Patient and community centered eHealth: Exploring eHealth barriers and facilitators for chronic disease self-management within British Columbia's immigrant Chinese and Punjabi seniors | Zibrik 2015 | Wrong setting |
| Patient interest in and barriers to telemedicine video visits in a multilingual urban safety-net system. | Khoong 2021 | Wrong patient population |
| Patient Perceptions of a Virtual Reality-Based System for Pulmonary Rehabilitation: A Qualitative Analysis. | Gabriel 2023 | barriers/facilitators not explored in a meaningful way |
| Patients and Caregivers Rate the PAIN Report It Wireless Internet-Enabled Tablet as a Method for Reporting Pain During End-of-Life Cancer Care. | Schoppee 2020 | barriers/facilitators not explored in a meaningful way |
| Patients' and healthcare providers' perceptions and experiences of telehealth use and online health information use in chronic disease management for older patients with chronic obstructive pulmonary disease: a qualitative study. | Jiang 2022 | Wrong patient population |
| Patients' Perceptions of Barriers and Facilitators to the Adoption of E-Hospitals: Cross-Sectional Study in Western China. | Li 2020 | barriers/facilitators not explored in a meaningful way |
| Perceived Benefits and Barriers of mHealth Mindfulness Use for Caregivers of Older Adults with Cognitive Impairment: A Qualitative Exploration. | Llaneza 2024 | Wrong patient population |
| Perception of Older Adults Toward Smartwatch Technology for Assessing Pain and Related Patient-Reported Outcomes: Pilot Study. | Manini 2019 | barriers/facilitators not explored in a meaningful way |
| Perceptions and use of technology to support self-management for older adults living with multiple health conditions within a care ecosystem | Murphy 2017 | barriers/facilitators not explored in a meaningful way |
| Perceptions of Home Telemonitoring Use Among Patients With Chronic Obstructive Pulmonary Disease: Qualitative Study | Lundell 2020 | Wrong patient population |
| Perceptions of older adults and health professionals about digital health tools for elder mistreatment screening | Choo 2021 | barriers/facilitators not explored in a meaningful way |
| Perceptions of older people in Ireland and Australia about the use of technology to address falls prevention. | Mackenzie 2020 | Wrong patient population |
| Perceptions of registered nurses on facilitators and barriers of implementing the AI-IoT-based healthcare pilot project for older adults during the covid-19 pandemic in South Korea. | Boo 2023 | Wrong setting |
| PERCEPTIONS OF TELEHEALTH BARRIERS AND BENEFITS DURING covid AMONG AN INTERDISCIPLINARY GROUP OF CARE PROVIDERS OF OLDER ADULTS WITH CANCER | Brintzenhofeszoc 2022 | Abstract |
| Perceptions Toward Internet-Based Delivery of Hearing Aids among Older Hearing-Impaired Adults. | Chandra 2016 | barriers/facilitators not explored in a meaningful way |
| Performance and Usability of Tablet Computers by Family Caregivers in the United States and China. | HongTao 2016 | barriers/facilitators not explored in a meaningful way |
| Perioperative telemonitoring of older adults with cancer: Can we connect them all? | Jonker 2020 | barriers/facilitators not explored in a meaningful way |
| Personalized Telehealth: Redesigning Complex Care Delivery for the 65+ During the covid Pandemic: a Survey of Patients, Caregivers, and Health-care Providers. | Nene 2023 | barriers/facilitators not explored in a meaningful way |
| Person-centered care and engagement via technology of residents with dementia in aged care facilities. | Goh 2017 | barriers/facilitators not explored in a meaningful way |
| PERSPECTIVE: Older Adults' Adoption of Technology: An Integrated Approach to Identifying Determinants and Barriers | Lee 2015 | Wrong study design |
| Perspectives of Older Patients with Blood Cancers, Caregivers, and Oncology Clinicians on Benefits and Barriers of Technology for Home Functional Assessment | Clancy 2022 | Abstract |
| Perspectives on assistive technology among older Norwegian adults receiving community health services | Halvorsrud 2021 | barriers/facilitators not explored in a meaningful way |
| Perspectives on the Barriers and Benefits of Diabetes Technology in Older Adults with Diabetes in the USA | DeCarlo 2021 | Wrong patient population |
| Persuasive eHealth to Support Home Rehabilitation of the Elderly After a Hip Operation: An Explorative Approach. | Alpay 2020 | barriers/facilitators not explored in a meaningful way |
| Persuasive eHealth to Support Home Rehabilitation of the Elderly After a Hip Operation: An Explorative Approach...30th Medical Informatics Europe Conference | ALPAY 2020 | Abstract |
| Potential barriers to the diffusion of technologies in community-dwelling older people: Data from the PRESTIGE study | Cella 2022 | Wrong study design |
| Potential legal issues when caring healthcare robot with communication in caring functions are used for older adult care | Yasuhara 2020 | Wrong study design |
| Prevalence of Health App Use Among Older Adults in Germany: National Survey | Rasche 2018 | barriers/facilitators not explored in a meaningful way |
| Primary care providers' perspectives on telemedicine in the pharmacologic management of older adults with Chronic Pain (CP) | Levine 2012 | Abstract |
| Promoting Advance Care Planning in Chinese Older Population Using a Gamification Approach: A Feasibility Trial | Chan 2023 | technology does not support care |
| Promoting user compliance with wearable hip protectors in older adults through education and technology...International Society for Gerontechnology 13th World Conference, October 24-26, 2022, Daegu, South Korea | Ho 2022 | Abstract |
| Qualitative investigation into a wearable system for chronic obstructive pulmonary disease: The stakeholders' perspective | Kayyali 2016 | barriers/facilitators not explored in a meaningful way |
| Reasons for refusing referrals and challenges to effectual engagement in tele-treatment for depression among low-income homebound older adults | Choi 2022 | Wrong patient population |
| Remote enrollment into a telehealth-delivering patient portal: Barriers faced in an urban population during the covid-19 pandemic | Francke 2022 | Wrong setting |
| Revolutionising home-based Multiple sclerosis care with Augmented reality Technology | Straukiene 2023 | Wrong study design |
| Rising to the Occasion: A National Nursing Home Study Documenting Attempts to Address Social Isolation During the covid-19 Pandemic. | Lapane 2023 | barriers/facilitators not explored in a meaningful way |
| Robot services for elderly with cognitive impairment: testing usability of graphical user interfaces | Granata 2013 | barriers/facilitators not explored in a meaningful way |
| Robotic Services Acceptance in Smart Environments With Older Adults: User Satisfaction and Acceptability Study. | Cavallo 2018 | barriers/facilitators not explored in a meaningful way |
| Robots and human dignity: a consideration of the effects of robot care on the dignity of older people | Sharkey 2014 | Wrong study design |
| Robots in care for older people: Opinions of potential end-users | Tobis 2017 | Abstract |
| Robots in Eldercare: How Does a Real-World Interaction with the Machine Influence the Perceptions of Older People?. | Tobis 2022 | barriers/facilitators not explored in a meaningful way |
| Robots to assist daily activities: Views of older adults with Alzheimer's disease and their caregivers. | Wang 2017 | barriers/facilitators not explored in a meaningful way |
| Role of Assistive Robots in the Care of Older People: Survey Study Among Medical and Nursing Students. | Åukasik 2020 | Wrong patient population |
| Rural older adults' intentions to use technology for monitoring mood states | Slosser 2019 | barriers/facilitators not explored in a meaningful way |
| Satisfying Product Features of a Fall Prevention Smartphone App and Potential Users' Willingness to Pay: Web-Based Survey Among Older Adults | Rasche 2018 | barriers/facilitators not explored in a meaningful way |
| Seniors' perspectives on the learning and using ICT: findings within the project AWAKE | LatviaUnivAgric 2013 | Wrong patient population |
| Sensors in support of aging-in-place: The good, the bad, and the opportunities. | Cook 2020 | Abstract |
| Service robots in elderly care at home: Users' needs and perceptions as a basis for concept development. | Pigini 2012 | barriers/facilitators not explored in a meaningful way |
| Significant challenges when introducing care robots in Swedish elder care | Johansson-Pajala 2020 | Wrong patient population |
| Skilled nursing resident adherence with wearable technology to offer safer mobility and decreased fall injuries. | Tarbert 2021 | technology does not support care |
| Smart moves for an aging population. | Josephsen 2021 | Wrong study design |
| Smart Nursing: The Use of Technology to Support Homecare Nurses with Their Care of the Elderly...17th International Conference of the Association for the Advancement of Assistive Technology in Europe, August 30- September 1, 2023, Aubervilliers, France | LEMMENS 2023 | Abstract |
| Smart Technologies in Social Housing: Methodology and First Results of the HOST Project Experimentation Activities | Biocca 2014 | Wrong study design |
| Social connections and participation among people with mild cognitive impairment: barriers and recommendations | Zhu 2023 | Wrong study design |
| Social Isolation and the Use of Technology in Caregiving Dyads Living With Dementia During covid-19 Restrictions. | Hoel 2022 | Wrong setting |
| Social Networks, New Technologies, and Wellbeing-An Interview Study on Factors Influencing Older Adults' Successful Ageing. | Betlej 2023 | Wrong patient population |
| Socially Assistive Robots for the Aging Population: Are We Trapped in Stereotypes? | ACM/IEEE 2014 | Wrong study design |
| Socially Assistive Robots in Aged Care: Ethical Orientations Beyond the Care-Romantic and Technology-Deterministic Gaze | Vandemeulebroucke 2021 | Wrong study design |
| Socially Assistive Robots: Measuring Older Adults' Perceptions | Beuscher 2017 | barriers/facilitators not explored in a meaningful way |
| Socio-technical barriers affecting large-scale deployment of AI-enabled wearable medical devices among the ageing population in China | Xing 2021 | barriers/facilitators not explored in a meaningful way |
| Socio-Technical Challenges in Implementation of Monitoring Technologies in Elderly Care | Kolkowska 2016 | Wrong study design |
| Strategies to Ensure Continuity of Care Using Telemedicine with Older Adults during covid-19: A Qualitative Study of Physicians in Primary Care and Geriatrics. | Chen 2022 | Wrong setting |
| Strategies to Implement Pet Robots in Long-Term Care Facilities for Dementia Care: A Modified Delphi Study. | Koh 2023 | barriers/facilitators not explored in a meaningful way |
| Studying the technological barriers and needs of people with dementia: A Wrong study designitative study | IEEE 2018 | barriers/facilitators not explored in a meaningful way |
| Successful implementation of new technologies in nursing care: a questionnaire survey of nurse-users | deVeer 2011 | barriers/facilitators not explored in a meaningful way |
| Successful treatment of an elderly patient with psychosis via video teleconferencing | Tan 2015 | Wrong study design |
| Suitability of Healthcare Robots for a Dementia Unit and Suggested Improvements | Robinson 2013 | Wrong study design |
| Supporting elderly homecare with smartwatches: advantages and drawbacks | Ehrler 2014 | barriers/facilitators not explored in a meaningful way |
| Supportive home health care technology for older adults: Attitudes and implementation. | Charness 2016 | barriers/facilitators not explored in a meaningful way |
| Tablets for deeply disadvantaged older adults: Challenges in long-term care facilities. | Cid 2020 | barriers/facilitators not explored in a meaningful way |
| Technological access barriers, telehealth use and health care visits in the early pandemic period | Maloney 2022 | barriers/facilitators not explored in a meaningful way |
| Technological health intervention in population aging to assist people to work smarter not harder: Qualitative study. | Chen 2018 | Wrong patient population |
| Technology acceptance and perceptions of robotic assistive devices by older adults - implications for exoskeleton design | Shore 2020 | barriers/facilitators not explored in a meaningful way |
| Technology acceptance and quality of life of the elderly in a telecare program | Chou 2013 | Wrong study design |
| Technology acceptance and quality of life of the elderly in a telecare program | Chou 2013 | Wrong study design |
| Technology and social care in a digital world: challenges and opportunities in the UK | Hamblin 2020 | Wrong study design |
| Technology and social media use by adult patients with intellectual and/or developmental disabilities | Crocitto 2020 | Wrong patient population |
| Technology Solutions to Support Care Continuity in Home Care: A Focus Group Study | Dowding 2018 | barriers/facilitators not explored in a meaningful way |
| Technology support to a telehealth in the home service: Qualitative observations. | Taylor 2016 | barriers/facilitators not explored in a meaningful way |
| Technology use & social isolation among older adults: A qualitative analysis | Fields 2020 | barriers/facilitators not explored in a meaningful way |
| Technology-based interprofessional collaboration in primary care for home rehabilitation of the older adults: A dutch exploratory study. | Alpay 2023 | Wrong patient population |
| Technology-based motivation support for seniors' physical activity-a qualitative study on seniors' and health care professionals' views | Ehn 2019 | barriers/facilitators not explored in a meaningful way |
| Teleassistance for frail elderly people: A usability and customer satisfaction study. | DeCola 2020 | barriers/facilitators not explored in a meaningful way |
| Telecare for the Elderly--Community Nurses' Experiences in Taiwan. | | barriers/facilitators not explored in a meaningful way |
| Telehealth at Home: Co-Designing a Smart Home Telehealth System. | HUNTER 2021 | barriers/facilitators not explored in a meaningful way |
| Telehealth for an Aging Population: How Can Law Influence Adoption Among Providers, Payors, and Patients? | Sklar 2020 | barriers/facilitators not explored in a meaningful way |
| Telehealth for persons with severe functional disabilities and their caregivers: facilitating self-care management in the home setting | Forducey 2012 | Wrong patient population |
| Telehealth survey for older adults and geriatricians during the covid-19 pandemic | Thomas 2021 | barriers/facilitators not explored in a meaningful way |
| Telehealth to Address Health Promotion & Social Isolation in Community Dwelling Seniors During a Public Health Emergency | Belanger 2023 | barriers/facilitators not explored in a meaningful way |
| Telehealth use and covid-19: Assessing older veterans' perspectives. | Weldon 2023 | Wrong setting |
| Telehealth was beneficial during covid?19 for older Americans: A qualitative study with physicians. | Goldberg 2021 | Wrong setting |
| Telemedicine and the ageing population | Otto 2015 | Wrong study design |
| Telemedicine in specialist outpatient care during covid-19: a qualitative study. | Sengupta 2024 | Wrong patient population |
| TELEMEDICINE TRAINING TO IMPROVE HEALTHCARE ACCESS FOR SENIOR LIVING COMMUNITY RESIDENTS IN LEWISTOWN, MONTANA | French 2021 | Abstract |
| Telemedicine use by older adults in a covid-19 epicenter | Truong 2021 | Abstract |
| Tele-operated android robot reminiscence group therapy and human coordinated RGT for older adults with dementia: A comparative study | Kase 2019 | barriers/facilitators not explored in a meaningful way |
| 'That is a place where I would want to go': investigating digital nature to enhance social wellbeing among older adults. | vanHouwelingen-Snippe 2023 | technology does not support care |
| That robot is not for me: Addressing stereotypes of aging in assistive robot design | IEEE 2016 | Wrong patient population |
| The acceptability of TV-based game platforms as an instrument to support the cognitive evaluation of senior adults at home | Costa 2017 | barriers/facilitators not explored in a meaningful way |
| The adoption of care robots in home careâ€”A survey on the attitudes of Finnish home care personnel. | Rantanen 2018 | barriers/facilitators not explored in a meaningful way |
| The Andragogical Perspectives of Older People's Interaction With Digital Game Technologies: Gameplay on Gesture and Touch-Based Platforms | Jali 2016 | barriers/facilitators not explored in a meaningful way |
| The barrier of using digital health in older people: A study in rural community, Thailand | Yodmai 2019 | Wrong study design |
| The Design of New Technology Supporting Wellbeing, Independence and Social Participation, for Older Adults Domiciled in Residential Homes and/or Assisted Living Communities | Cahill 2018 | barriers/facilitators not explored in a meaningful way |
| The digital divide among low-income homebound older adults: Internet use patterns, eHealth literacy, and attitudes toward computer/Internet use. | Choi 2013 | barriers/facilitators not explored in a meaningful way |
| The Effect of Chronic Diseases on the Use of Health Technology and Digital Services in the Elderly Population in Finland...European Federation for Medical Informatics Special Topic Conference 2020 (Virtual), 26-27 November 2020. | MIELONEN 2020 | Abstract |
| The environmental factors that influence technology adoption for older adults with age-related vision loss | McGrath 2019 | Wrong patient population |
| The experience of older persons with mental health conditions who interact with healthcare robots and nurse intermediaries: The qualitative case studies. | Tanioka 2021 | Wrong patient population |
| The feasibility and acceptability of smart home technology using the Howz system for people with stroke. | Rogerson 2020 | barriers/facilitators not explored in a meaningful way |
| The iMHere 2.0 System for Family Caregivers of Older Adults: A Focus Group. | Hu 2023 | barriers/facilitators not explored in a meaningful way |
| The Impact of covid-19 on Older Adults' Perceptions of Virtual Care: Qualitative Study. | Abdallah 2022 | Wrong setting |
| The incessant watch: Using technology in the care of the aging patient | Robertson 2020 | barriers/facilitators not explored in a meaningful way |
| The Modified and Extended Hospital Elder Life Program: A remote model of care to expand delirium prevention. | Fong 2023 | Wrong patient population |
| The Perspective of Nurses and Healthcare Providers on the use of Television Videos with People with Moderate to Severe Dementia. | Hung 2024 | barriers/facilitators not explored in a meaningful way |
| The place and role of Skype consultancies among palliative patients and the impact of this type of care on a quality of life, pain, anxiety and depression symptoms assessment in home hospice care patient | Kieszkowska-Grudny 2016 | Abstract |
| The potential of technology supports for healthy aging: From apps to robots | Rogers 2015 | Abstract |
| The role of a socially assistive robot in enabling older adults with mild cognitive impairment to cope with the measures of the covid-19 lockdown: A qualitative study. | Van Assche 2023 | Wrong setting |
| The role of combinatorial health technologies in supporting older people with long-term conditions: Responsibilisation or co-management of healthcare? | Varey 2021 | barriers/facilitators not explored in a meaningful way |
| The Role of Conversational AI in Ageing and Dementia Care at Home: A Participatory Study | Lima 2023 | Wrong patient population |
| The Role of Health Status in Older Adults' Perceptions of the Usefulness of eHealth Technology | Best 2015 | barriers/facilitators not explored in a meaningful way |
| The Role of Healthcare Robotics in Providing Support to Older Adults: a Socio-ecological Perspective | Mois 2020 | Wrong study design |
| The Role of Psychological Factors in Older Adults' Readiness to Use eHealth Technology: Cross-Sectional Questionnaire Study. | Knapova 2020 | barriers/facilitators not explored in a meaningful way |
| The Usability and Impact of a Low-Cost Pet Robot for Older Adults and People With Dementia: Qualitative Content Analysis of User Experiences and Perceptions on Consumer Websites. | Koh 2022 | Wrong study design |
| The use of accelerometer-based wearable activity monitors in clinical settings: current practice, barriers, enablers, and future opportunities. | Maher 2021 | Wrong setting |
| The use of information and communication technologies to support working carers of older people - a qualitative secondary analysis. | Andersson 2016 | Wrong study design |
| The Use of Smart Speakers in Care Home Residents: Implementation Study. | Edwards 2021 | technology does not support care |
| The use of technology to address loneliness and social isolation among older adults: the role of social care providers. | Gray 2024 | Wrong study design |
| The Use of Technology-Enabled Care (TEC) in Patients with Parkinson's Disease: An Italian Survey | DiNuzzo 2018 | barriers/facilitators not explored in a meaningful way |
| 'They just came with the medication dispenser'- a qualitative study of elderly service users' involvement and welfare technology in public home care services. | GlomsÃ¥s 2021 | barriers/facilitators not explored in a meaningful way |
| Together, at a distance: experiences with a novel technology for social contact among older people and their relatives in Norway during the covid-19 pandemic. | Badawy 2023 | barriers/facilitators not explored in a meaningful way |
| Toward improved homecare of frail older adults: A focus group study synthesizing patient and caregiver perspectives | McDonald 2021 | barriers/facilitators not explored in a meaningful way |
| Toward usable and acceptable robot interfaces for the elderly: The robot-era project experience | Broz 2015 | Abstract |
| Transition and Sustainability of an Online Care Model for People With Parkinson's Disease in Response to the covid-19 Pandemic. | Ketigian 2021 | Wrong setting |
| Types of Telehealth services preferred by geriatric patients during the covid-19 pandemic | Nguyen 2022 | Abstract |
| Understanding Older Adult's Technology Adoption and Withdrawal for Elderly Care and Education: Mixed Method Analysis from National Survey | Chiu 2017 | Wrong patient population |
| Understanding Older People's Readiness for Receiving Telehealth: Mixed-Method Study. | vanHouwelingen 2018 | Wrong patient population |
| Understanding the Experience of Geriatric Care Professionals in Using Telemedicine to Care for Older Patients in Response to the covid-19 Pandemic: Mixed Methods Study. | Chen 2022 | Wrong setting |
| Usability and Acceptability of a Palliative Care Mobile Intervention for Older Adults With Heart Failure and Caregivers: Observational Study. | Villalobos 2022 | barriers/facilitators not explored in a meaningful way |
| Usability evaluation for the Amulet Wearable Device in rural older adults with obesity. | Batsis 2018 | Wrong patient population |
| Usability evaluation of assistive technologies through qualitative research focusing on people with mild dementia. | Asghar 2018 | barriers/facilitators not explored in a meaningful way |
| Use of a mobile app by older people in an integrated care setting. | Azaria 2020 | Wrong study design |
| Use of technology by people with dementia and informal carers during covid-19: A cross-country comparison | Chirico 2022 | Wrong setting |
| Use of video visits in home-based palliative care, qualitative perceptions from clinicians | Macias 2020 | barriers/facilitators not explored in a meaningful way |
| Use of WhatsApp by older adults screened for depression in socioeconomically deprived areas of Guarulhos, São Paulo State, Brazil: challenges and possibilities for telehealth | Moretti 2023 | Wrong patient population |
| User acceptance of location-tracking technologies in health research: Implications for study design and data quality. | Hardy 2018 | barriers/facilitators not explored in a meaningful way |
| User experiences of older adults navigating an online database of community-based physical activity programs | Lowndes 2023 | barriers/facilitators not explored in a meaningful way |
| User perspectives on emotionally aligned social robots for older adults and persons living with dementia. | Dosso 2022 | Wrong patient population |
| User-Centered Development of a Web Platform Supporting Community-Based Health Care Organizations for Older Persons in Need of Support: Qualitative Focus Group Study. | Biehl 2021 | barriers/facilitators not explored in a meaningful way |
| User-centered requirements engineering to manage the fuzzy front-end of open innovation in e-health: A study on support systems for seniors' physical activity. | Ehn 2021 | barriers/facilitators not explored in a meaningful way |
| Using computers and the internet: The experiences of older adults in assisted living communities. | Winstead 2015 | barriers/facilitators not explored in a meaningful way |
| Using eHealth Technologies: Interests, Preferences, and Concerns of Older Adults | Ware 2017 | barriers/facilitators not explored in a meaningful way |
| Using HIT to deliver integrated care for the frail elderly in the UK: current barriers and future challenges | Waterson 2012 | barriers/facilitators not explored in a meaningful way |
| Using HIT to deliver integrated care for the frail elderly in the UK: current barriers and future challenges. | Soares 2012 | barriers/facilitators not explored in a meaningful way |
| Using Information and Communication Technologies to Promote Healthy Aging in Costa Rica: Challenges and Opportunities | Rojas 2016 | Wrong study design |
| Using Internet technologies in rural communities to access services: The views of older people and service providers | Hodge 2017 | barriers/facilitators not explored in a meaningful way |
| Using mobile health and the impact on health-related quality of life: Perceptions of older adults with cognitive impairment | Christiansen 2020 | barriers/facilitators not explored in a meaningful way |
| Using Teams, Real-Time Information, And Teleconferencing To Improve Elders' Hospital Care. | Meyer 2011 | barriers/facilitators not explored in a meaningful way |
| Using Technology for Health Promotion in Older Adults with Cognitive Impairment | Shah 2023 | Abstract |
| Using technology to improve care of older adults. | Chau 2018 | Wrong study design |
| Using the Technology Acceptance Model to explore community dwelling older adults' perceptions of a 3D interior design application to facilitate pre-discharge home adaptations | Money 2015 | barriers/facilitators not explored in a meaningful way |
| Using the Technology Acceptance Model to Explore User Experience, Intent to Use, and Use Behavior of a Patient Portal Among Older Adults With Multiple Chronic Conditions: Descriptive Qualitative Study. | Portz 2019 | Wrong patient population |
| Using Wearable Sensors to Measure Goal Achievement in Older Veterans with Dementia. | Freytag 2022 | barriers/facilitators not explored in a meaningful way |
| Video Calls as a Replacement for Family Visits During Lockdowns in Aged Care: Interview Study With Family Members. | Kelly 2023 | Wrong setting |
| Video Telehealth Occupational Therapy Services for Older Veterans: National Survey Study. | Gately 2021 | Wrong study design |
| Virtual collaborative creative engagement in a pandemic world: creative connection for older adults with lived experience of dementia. | Henderson 2023 | technology does not support care |
| Voices of Experience: What Do Low-Income Older Adults Tell Us About Mobility, Technology, and Social Participation? | | Wrong patient population |
| Wear It or Fear It Exploration of Drivers & Barriers in Smartwatch Acceptance by Senior Citizens | Askari 2018 | Abstract |
| Wearable and ambient sensor technology in geriatric medicine and rehabilitation-examples, challenges and lessons learned | Marschollek 2016 | Wrong study design |
| Weaving Social Connectivity into the Community Fabric: Exploring Older Adult's Relationships to Technology and Place. | Paulovich 2022 | technology does not support care |
| What Are Real Problems of Older Adults and How to Solve It With Smart Home Devices? | IEEE 2018 | barriers/facilitators not explored in a meaningful way |
| What are the key contextual factors when preparing for successful implementation of assistive living technology in primary elderly care? A case study from Norway | Gjestsen 2017 | Wrong study design |
| What is 'care quality' and can it be improved by information and communication technology? A typology of family caregivers' perspectives. | Leslie 2021 | barriers/facilitators not explored in a meaningful way |
| What is 'care quality' and can it be improved by information and communication technology? A typology of family caregivers' perspectives. | Leslie 2020 | barriers/facilitators not explored in a meaningful way |
| What it Takes to Successfully Implement Technology for Aging in Place: Focus Groups With Stakeholders. | Peek 2016 | barriers/facilitators not explored in a meaningful way |
| Why is it difficult to implement e-health initiatives? A qualitative study. | Murray 2011 | Wrong patient population |
| Wrong study designifying beliefs regarding telehealth: Development of the Whole Systems Demonstrator Service User Technology Acceptability Questionnaire. | Hirani 2017 | barriers/facilitators not explored in a meaningful way |
| You Can Teach an Old Dog New Tricks: A Qualitative Analysis of How Residents of Senior Living Communities May Use the Web to Overcome Spatial and Social Barriers | Winstead 2013 | barriers/facilitators not explored in a meaningful way |
| Learning From Older Adults to Promote Independent Physical Activity Using Mobile Health (mHealth). | Nebeker 2021 | Wrong study design |
| If You Give Them Away, It Still May Not Work: Challenges to Video Telehealth Device Use Among the Urban Homebound. | Liozos 2023 | Wrong study design |
| Connecting the disconnected: pharmacist performed virtual care visits for older adults with technological support | Hawley 2023 | Wrong study design |

**Supplementary Table S9: MEDLINE search strategy**

| **Search step** | **Search Syntax** |
| --- | --- |
| 1 | exp Information Technology/ or exp Biomedical Technology/ or exp Technology/ or exp Wireless Technology/ or exp Digital Technology/ or exp Remote Sensing Technology/ or exp Technology Assessment, Biomedical/ |
| 2 | (Technolog* or Sensor* or camera* or ehealth* or e-health or smart* or computer* or robot* or mobile app* or medical informatic* or health information exchange or health smart cards or mobile health or electronic health or electronic care or personal digital assistant or patient monitor or monitor or wireless or information tech* or wearable or mobile tech* or personal alarm).tw. |
| 3 | 1 or 2 |
| 4 | exp Aged/ or exp Aging/ |
| 5 | (older adult* or older person* or older people or elderly or later life or senior*).tw. |
| 6 | 4 or 5 |
| 7 | (barrier* or facilitat* or hinder* or block* or obstacle* or restrict* or restrain* or obstruct* or impede* or hindrance* or encourag* or optimiz* or optimis* or challenge* or enable*).tw. |
| 8 | exp Uncompensated Care/ or exp Secondary Care/ or exp "Hospice and Palliative Care Nursing"/ or exp Adult Day Care Centers/ or exp Self Care/ or exp Respite Care/ or exp "Delivery of Health Care"/ or exp Primary Health Care/ or exp Home Care Agencies/ or exp Process Assessment, Health Care/ or exp Nursing Care/ or exp Home Care Services/ or exp Secondary Care Centers/ or exp Primary Care Nursing/ or exp Palliative Care/ or exp "Delivery of Health Care, Integrated"/ or exp Hospice Care/ or exp Long-Term Care/ |
| 9 | (Care or home care or care home or long term care or care escalation or care home admission or domiciliary care or carer* or paid care* or unpaid care or formal care or informal care or electronic care or nursing home or community care or assisted living or in home care or in-home care or palliative care or retirement village or at home care* or retirement home or care in the community or social support or support living or help at home or home adaption or sheltered or family practice or general practice or housing).tw. |
| 10 | 8 or 9 |
| 11 | 3 and 6 and 7 and 10 |
| 12 | limit 11 to yr="2011 -Current" Ovid MEDLINE(R) <1946 to March Week 1 2024>  Restrictions: English only |
